# MEF2A transcriptionally upregulates the expression of ZEB2 and CTNNB1 in colorectal cancer to promote tumor progression

**DOI:** 10.1038/s41388-021-01774-w

**Published:** 2021-04-16

**Authors:** Qing Xiao, Yaqi Gan, Yimin Li, Lili Fan, Jiaqi Liu, Pengyan Lu, Jiaxin Liu, Aoao Chen, Guang Shu, Gang Yin

**Affiliations:** 1 Department of Pathology, Xiangya Hospital, School of Basic Medical Sciences, Central South University, Changsha, China; 2 Department of Immunology, School of Basic Medical Sciences, Central South University, Changsha, China; 3 Department of Histology and Embryology, School of Basic Medical Sciences, Central South University, Changsha, China; 4 China-Africa Research Center of Infectious Diseases, School of Basic Medical sciences, Central South University, Changsha, China

**Keywords:** Colorectal cancer, Transcription

## Abstract

Colorectal cancer (CRC) is one of the leading cancers worldwide, accounting for high morbidity and mortality. The mechanisms governing tumor growth and metastasis in CRC require detailed investigation. The results of the present study indicated that the transcription factor (TF) myocyte enhancer factor 2A (MEF2A) plays a dual role in promoting proliferation and metastasis of CRC by inducing the epithelial-mesenchymal transition (EMT) and activation of WNT/β-catenin signaling. Aberrant expression of MEF2A in CRC clinical specimens was significantly associated with poor prognosis and metastasis. Functionally, MEF2A directly binds to the promoter region to initiate the transcription of *ZEB2* and *CTNNB1*. Simultaneous activation of the expression of EMT-related TFs and Wnt/β-catenin signaling by MEF2A overexpression induced the EMT and increased the frequency of tumor formation and metastasis. The present study identified a new critical oncogene involved in the growth and metastasis of CRC, providing a potential novel therapeutic target for CRC intervention.

## Introduction

Colorectal cancer (CRC) is the most common malignancy in the digestive system and has high incidence and mortality [1]. Metastasis is the main cause of CRC-related mortality. The mechanisms governing tumor growth and metastasis have been extensively investigated but remain poorly understood.

The myocyte enhancer factor 2 (MEF2) family is composed of mammalian transcription factors (TFs) MEF2A, MEF2B, MEF2C, and MEF2D [2]. MEF2s, especially MEF2A and MEF2C, play an important role in embryonic development, controlling cytotrophoblast invasion, differentiation, B-cell development, and cardiogenesis [3–5]. Abnormal expression or mutation of MEF2 is closely associated with the progression of various tumors, such as large B-cell lymphoma [6], leukemia [7], and certain solid tumors (hepatocellular carcinoma [8] and CRC [9]). MEF2B, MEF2C, and MEF2D are predominantly involved in tumor proliferation [10], migration, and invasion [11] and in tumor immunity [12]. The structure of the *MEF2A* gene is highly homologous to that of the *MEF2C* gene, and MEF2A plays a key role in embryonic development similar to MEF2C; however, MEF2A is poorly characterized and has been rarely studied in the context of tumor progression.

Only a few recent reports investigated the function of MEF2A in tumors [13–15]. A recent study indirectly suggested that MEF2A can promote metastasis of CRC [16]. However, potential mechanism of MEF2A involvement in CRC proliferation and metastasis remains unclear.

The activation of the WNT/β-catenin signaling pathway is an important event in CRC initiation and progression [17]. On the other hand, the activation of the WNT/β-catenin signaling pathway can effectively induce the epithelial-mesenchymal transition (EMT) by regulating the expression of EMT-related TFs [18]. The EMT provides for the acquisition of metastatic properties by tumor cells to initiate metastasis in many types of malignancies including CRC [19, 20]. MEF2A positively regulates WNT signaling by directly upregulating Gtl2-Dio3 miRNAs during skeletal muscle regeneration [21]. However, few studies have reported the relationships between MEF2A and activation of WNT signaling in tumors.

The present study demonstrated that CRC patients with a higher level of MEF2A have shorter survival time and that tumors in this group of patients have higher incidence of distant metastases. The results of the present study demonstrated that MEF2A plays a role in the progression of CRC by upregulating the expression of ZEB2 and β-catenin to enhance the EMT and the activity of the WNT/β-catenin signaling pathway.

## Results

### MEF2A was upregulated in CRC tissues and was associated with poor prognosis

**Fig. 1:**
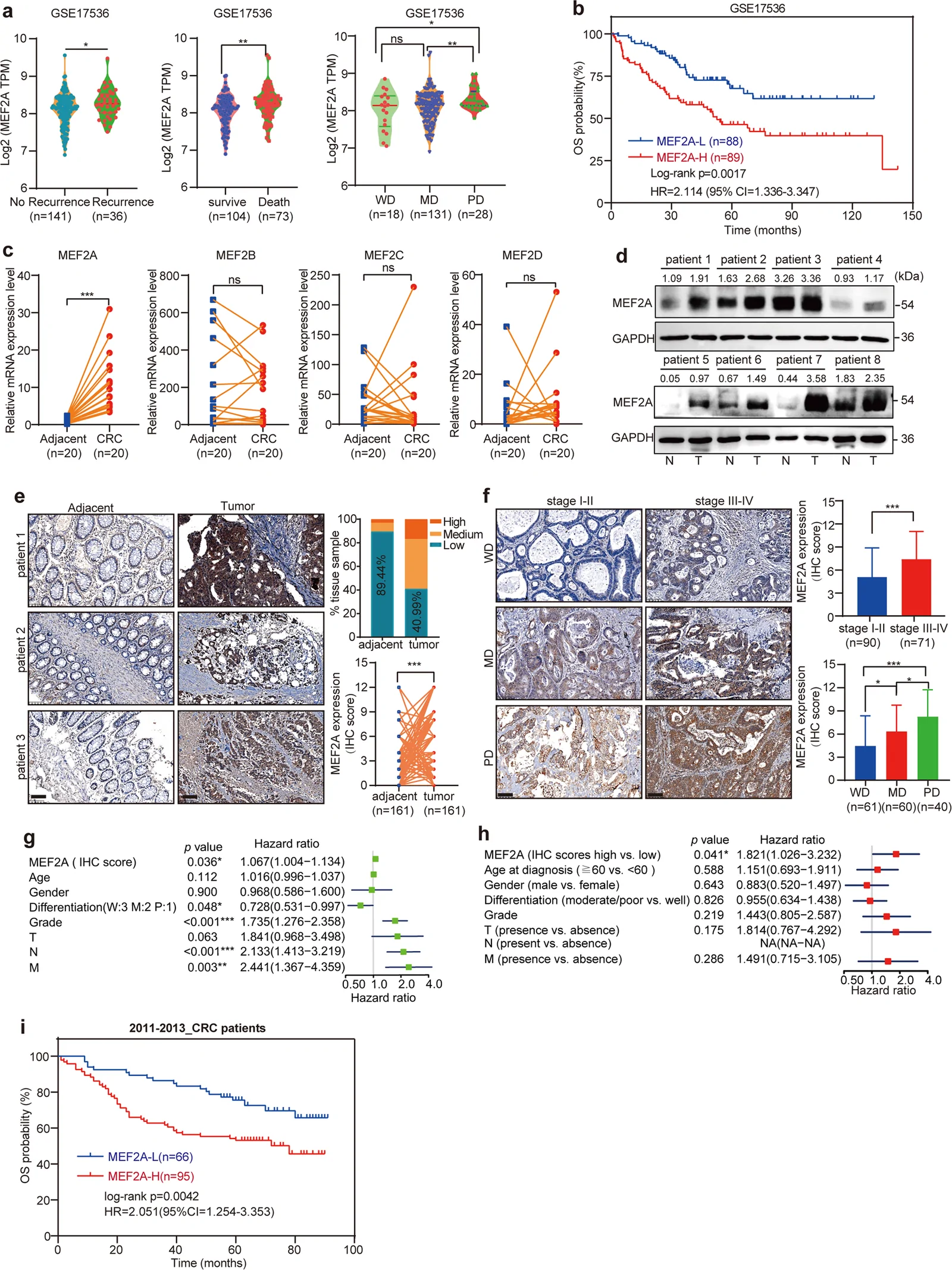
MEF2A was expressed at a high level in human CRC tissues and correlated with unfavorable prognosis. **a** MEF2A expression in the GSE17536 dataset (*n* = 177) (Student’s *t* test). **b** Kaplan–Meier curve showing the OS probability of CRC patients from the GSE17536 dataset expressing low or high levels of *MEF2A* mRNA (log-rank test). OS was defined as the interval between the date of surgery and the date of death or last follow-up. **c** qPCR analysis of the mRNA levels of MEF2s in 20 pairs of matched CRC and adjacent normal tissues (paired *t* test). **d** MEF2A protein levels in fresh CRC (T) and adjacent normal samples (N) (*n* = 8) detected by western blotting. **e** Representative IHC images of CRC tissues and corresponding normal tissues stained with an MEF2A antibody (*n* = 161). Magnification: 100×. Scale bar: 100 µm. **f** Representative IHC images of MEF2A staining of CRC tissues of various stages and histological differentiation. Magnification: 100×. Scale bar: 100 µm. WD well differentiated, MD moderate differentiated, PD poor differentiated. Univariate (**g**) and multivariate Cox proportional hazard analysis (**h**) of risks related to the prognosis. **i** Kaplan–Meier curve showing the associations of OS of the patients with CRC and MEF2A expression (MEF2A-L, *n* = 66; MEF2A-H, *n* = 95; log-rank test). OS overall survival, HR hazard ratio, CI confidence interval. All experiments were repeated independently three times. The values are shown as the mean ± SD.

Initially, a correlation between MEF2s expression and overall survival (OS) of CRC patients was analyzed using the colon and rectal cancer dataset of TCGA to determine the role of MEF2s in CRC. The results indicated that patients with higher levels of MEF2A or MEF2C, but not MEF2B and MEF2D, had shorter OS (Supplementary Fig. S1a). Then, we investigated an association of the expression of MEF2s mRNA with clinical feature and prognosis using a representative microarray Gene Expression Omnibus (GEO) dataset of human CRC samples, GSE17536 [22]. In GSE17536, higher levels of MEF2A and MEF2C were detected in patients with recurrence and death events (Fig. 1a, Supplementary Fig. S1b). Survival analysis using GES17536 showed that higher levels of MEF2A and MEF2C were significantly associated with shorter OS (Fig. 1b, Supplementary Fig. S1c). Importantly, only MEF2A was significantly upregulated in poor differentiated tissues (Fig. 1a). These results suggested that MEF2s may be important for CRC development. Hence, we assayed the levels of *MEF2s* mRNA in intact CRC tissue samples and corresponding normal tissue samples, which were collected at the Department of General Surgery, Xiangya Hospital. The results showed that only the expression of *MEF2A* mRNA was consistently higher in the tumor tissues than that in adjacent tissues, and statistical analysis indicated significant differences (Fig. 1c, Supplementary Fig. S1d). In agreement with the results of qPCR, the levels of the MEF2A protein were elevated in tumor tissue samples compared to that in the control samples (Fig. 1d). These results suggested that MEF2A is more to play an important role in the progression of CRC than other MEF2 member. To verify the effects of MEF2A on CRC progression, a total of 161 CRC samples and paired colorectal mucosa samples were collected and subjected to immunohistochemistry (IHC) staining. The results of the staining indicated that 59.01% of the tumor tissue samples and 10.56% of adjacent tissue samples showed positive staining for MEF2A, and the staining intensity was stronger in the tumor tissues than that in adjacent tissues (Fig. 1e). Moreover, the staining intensity of MEF2A was considerably stronger in poorly differentiated and late-stage tumor tissues than that in well-differentiated and early-stage tumor tissues (Fig. 1f). The IHC score of MEF2A was used to divide patients into two groups to analyze correlations between MEF2A expression and clinical and pathological features. High levels of MEF2A in CRC patients were correlated with poor histological differentiation, late TNM stage, lymph node metastasis, distant metastasis, and low OS (Table 1, Fig. 1i). Univariate Cox proportional hazard analysis showed that high levels of MEF2A, poor differentiation of the tumor tissues, and tumor grade were significantly associated with unfavorable prognostic risk (Fig. 1g). Multivariate analysis demonstrated that MEF2A may be an independent predictor of CRC prognosis (Fig. 1h). Overall, these results demonstrated that MEF2A may play an important role in the acceleration of tumor progression.

**Table 1:** Association between MEF2A levels in tumor tissues and clinicopathological characteristics of CRC patients.

Variable	MEF2A expression		^*A*^*P* value
	Low (*N* = 66)	High (*N* = 95)	
Sex, *N* (%)			0.745
M	40 (40.0)	60 (60.0)	
F	26 (42.6)	35 (57.4)	
Age at diagnosis, *N* (%)			0.747
≧60	27 (39.1)	42 (60.9)	
<60	39 (42.4)	53 (57.6)	
Histological differentiation, *N* (%)			**0.000**
Well	37 (60.7)	24 (39.3)	
Moderate	21 (35.0)	39 (65.0)	
Poor	8 (20.0)	32 (80.0)	
TNM Stage, *N* (%)			**0.010**
I-II	45 (50.0)	45 (50.0)	
III-IV	21 (29.6)	50 (70.4)	
Lymph node metastasis, *N* (%)			**0.010**
Absent	45 (50.0)	45 (50.0)	
Present	21 (29.6)	50 (70.4)	
Distant metastasis, *N* (%)			**0.005**
Absent	64 (44.8)	79 (55.2)	
Present	2 (11.1)	16 (88.9)	
Overall survival, *N* (%)			**0.009**
Alive	47 (49.5)	48 (50.5)	
Dead	19 (28.8)	47 (71.2)	

Samples: The Xiangya Hospital (Changsha, China); ^A^χ^2^ test; *n* = 161.

Bold values indicate statistical significance *P* < 0.05.

### MEF2A enhanced the proliferation, migration, and invasion of CRC cells in vitro

**Fig. 2:**
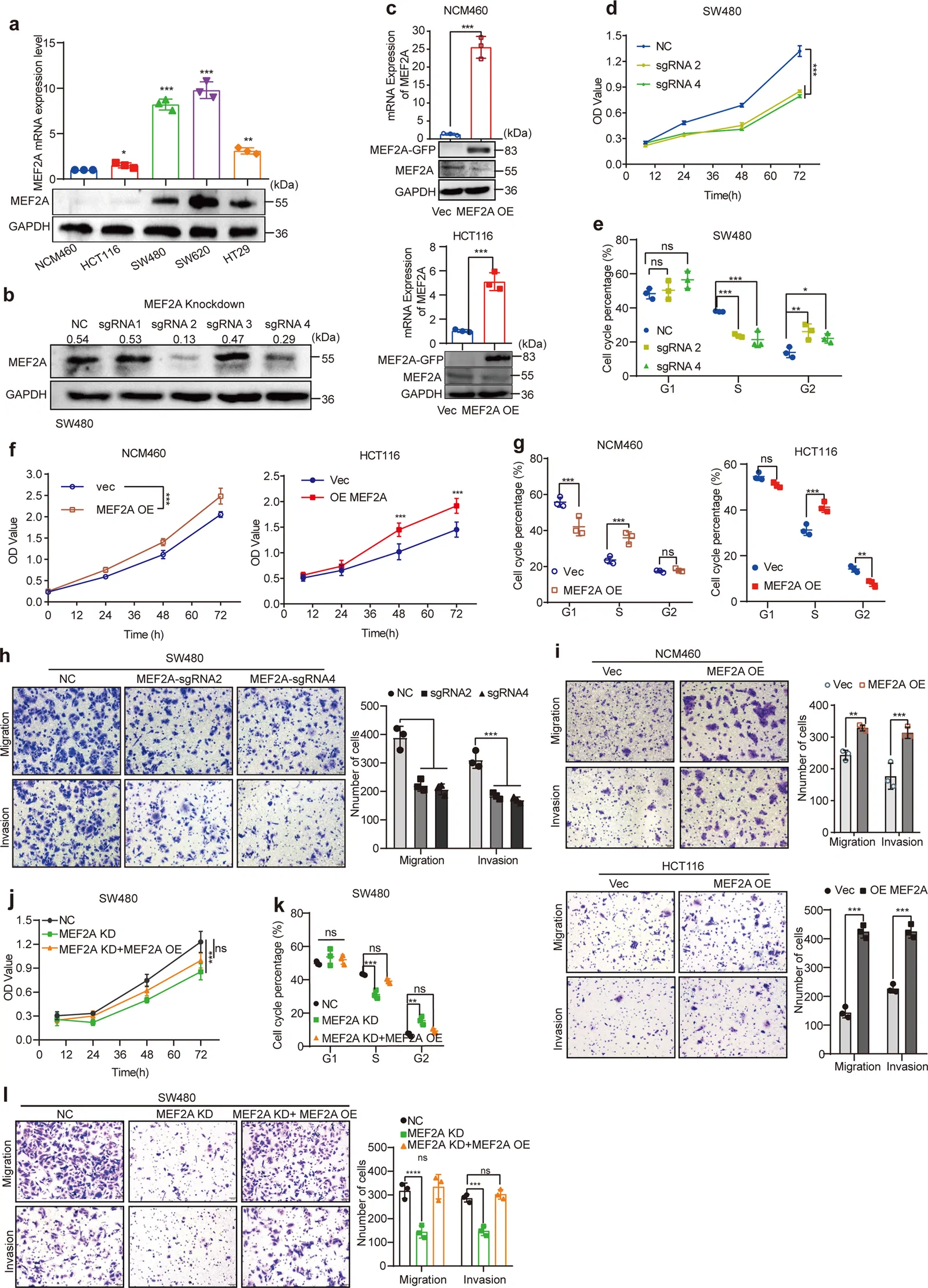
MEF2A promoted the biological function of CRC cells in vitro. **a** The levels of MEF2A protein and mRNA in a normal human colonic cell line and in the CRC cell lines. **b**, **c** Efficiency of MEF2A knockdown in SW480 cells. NC negative control. **b** Efficiency of MEF2A overexpression in NCM460 and HCT116 cells. Vec vector, MEF2A-GFP fusion protein (**c**). MTS assay, flow cytometry, and Transwell assay were used to measure the rate of cell growth (**d**, **f**), cell cycle distribution (**e**, **g**), and cell motility (**h**, **i**) after knockdown and overexpression of MEF2A. Detection of the rate of cell growth (**j**), cell cycle distribution (**k**), and cell motility (**l**) in the negative control, MEF2A knockdown, or MEF2A rescue groups. All experiments were repeated independently three times. The values are shown as the mean ± SD. The data in this figure were analyzed by Student’s *t* test.

The effects of downregulation or overexpression of MEF2A on malignant phenotypes of CRC cells and normal colonic epithelial cells were investigated. Detection of the expression of MEF2A in normal colonic epithelial cells (NCM460) and human CRC cell lines demonstrated that MEF2A was expressed at a high level in CRC cells (Fig. 2a). Then, MEF2A was knocked down (KD) and overexpressed in CRC and NCM460 cells, respectively (Fig. 2b, c). The results showed that silencing MEF2A markedly inhibited the proliferation of SW480 cells by causing the G2/M phase arrest (Fig. 2d, e), and MEF2A overexpression (OE) substantially promoted the growth of both NCM460 and HCT116 cells by increasing the subpopulations of the cells in the S-phase of the cell cycle (Fig. 2f, g). However, knockdown of MEF2A had only a few effects on apoptosis (Supplementary Fig. S2a). Moreover, downregulation of MEF2A resulted in a reduction in the migration and invasion of SW480 cells (Fig. 2h), and these effects were enhanced by MEF2A OE in NCM460 and HCT116 cells (Fig. 2i). These results were closely replicated by knockdown of MEF2A in SW480 and SW620 cells using shRNA or overexpression of MEF2A in SW480 cells (Supplementary Fig. S2b–i). Moreover, we ensured that the influence of MEF2C on the function of MEF2A was eliminated by knocking down MEF2C alone and by knocking down MEF2C with simultaneous overexpression of MEF2A. The results showed that MEF2C had only a few effects on the proliferation and migration of SW480 cells and had no synergistic effect with MEF2A (Supplementary Fig. S2j–k). Furthermore, we ectopically expressed MEF2A in stable MEF2A knockdown cells; the data indicated that impaired proliferation, migration, and invasion of CRC cells were restored (Fig. 2j–l). These results provide convincing evidence that MEF2A plays an instrumental role in the growth and metastasis of CRC in vitro.

### MEF2A facilitated tumor growth and metastasis of CRC in vivo

**Fig. 3:**
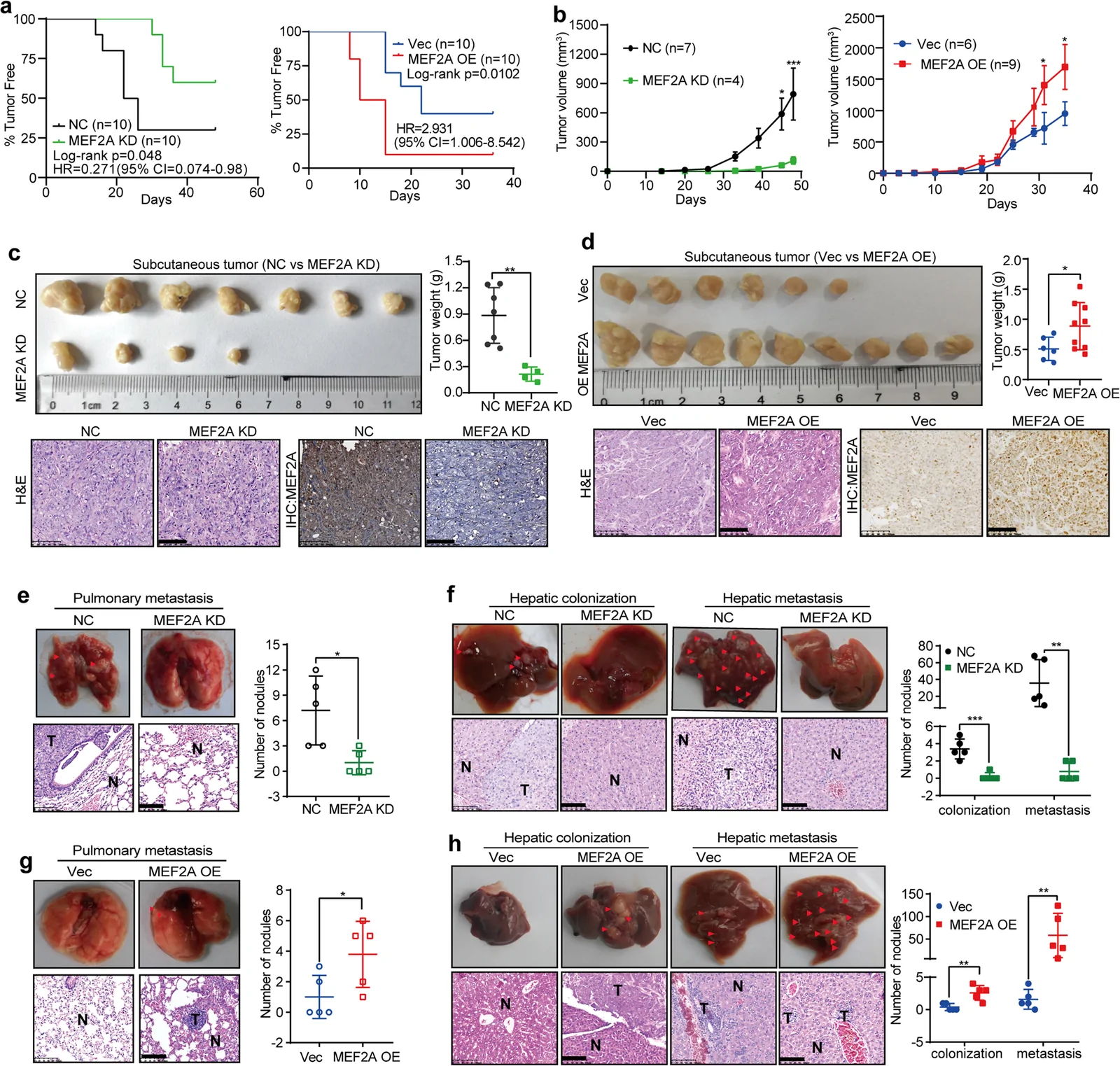
MEF2A promoted tumor growth and metastasis in vivo. **a** Kaplan–Meier plots showing the tumor-free survival of mice subcutaneously injected with tumor cells (log-rank test). **b** The growth rate of the tumors derived from SW480-MEF2A KD and SW480-NC cells or from HCT116-MEF2A OE and HCT116-vec cells (one-way ANOVA). Tumor growth was monitored every two days. The values are shown as the mean ± SEM. Representative tumor images, HE and IHC staining images of the MEF2A knockdown (**c**) and overexpression (**d**) groups. Magnification: 100×. Scale bar: 100 µm (Student’s *t* test). Representative images of pulmonary metastasis in the NC and MEF2A KD groups (**e**) and in the control and MEF2A OE groups (**g**) (Student’s *t* test). Representative images of hepatic colonization and metastatic lesions in the NC and MEF2A KD groups (**f**) and in the control and MEF2A OE groups (**h**) (Student’s *t* test). Tumor nodules formed in the tail vein injection group were defined as metastatic lesions and those formed in the intraperitoneal injection group were defined as colonization lesions. N normal, T tumor. All experiments were independently repeated three times.

A subcutaneous xenograft model was used to explore the effects of MEF2A on tumor growth. The results demonstrated that silencing MEF2A decreased the frequency of tumor formation and tumor growth rate, and overexpression of MEF2A achieved the opposite effects (Fig. 3a, b). Thus, the tumor volume and weight in animals of the high MEF2A expression group were considerably higher than those in the low MEF2A expression group (Fig. 3c, d). Effects of MEF2A on tumor cell colonization and dissemination were assessed in two metastasis models. Mice were injected into the abdominal cavity or tail vein with CRC cells with stable knockdown or overexpression of MEF2A. Silencing MEF2A dramatically reduced the number of hepatic and pulmonary metastases (Fig. 3e, f). The degree of mesenteric and diaphragmic colonization in the knockdown group was lower than that in the control group (Supplementary Fig. S3a, b). On the other hand, mice in the MEF2A OE group manifested an increase in mesenteric, diaphragmic, and hepatic colonization and a higher number of pulmonary and hepatic metastatic lesions (Fig. 3g, h, Supplementary Fig. S3c, d). These data indicated that high levels of MEF2A enhanced the malignant progression of CRC cells in vivo.

### Ectopic expression of MEF2A induced the EMT in CRC cells

**Fig. 4:**
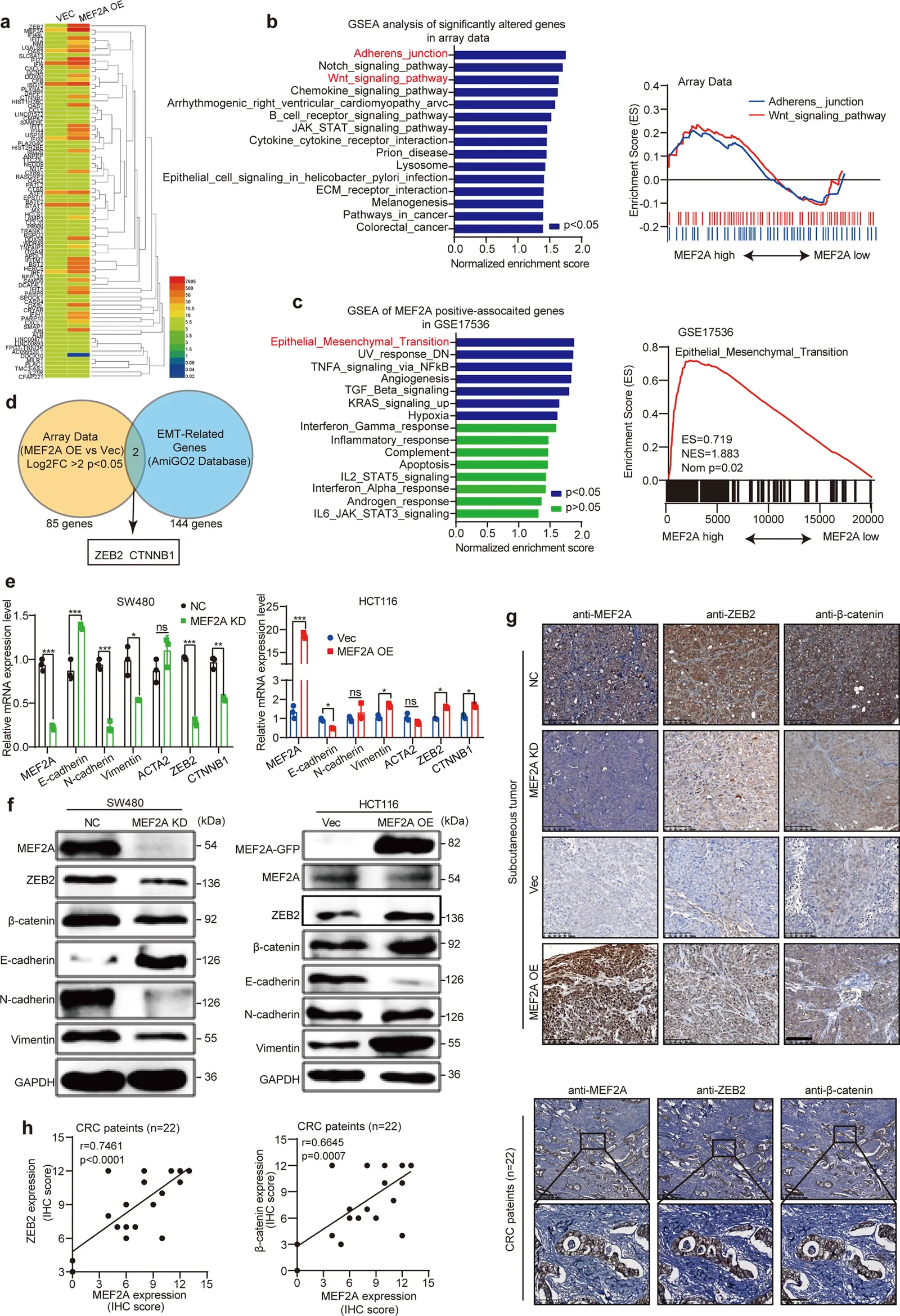
MEF2A participated in the EMT. **a** A cluster heatmap of expression profiles of mRNAs in HCT116-Vec and HCT116-MEF2A OE cells (|log2FC| > 2; *p* < 0.05). **b** GSEA of significantly altered genes in the microarray dataset (in HCT116- MEF2A OE vs HCT116-vec cells) (left). GSEA showed a correlation between *MEF2A* and EMT-related pathways (right). ES enrichment score, GSEA gene set enrichment analysis. **c** GSEA identified MEF2A positive-associated gene sets in the GSE17536 dataset (left) and indicated a positive correlation between the expression of MEF2A mRNA and the expression of the genes that participate in the EMT (right). NES normalized enrichment score. **d** Comparison of the genes upregulated in the array data (log2FC > 2; *p* < 0.05) with EMT-related gene in the AmiGO2 database (human species). **e**, **f** qPCR and western blot analysis of EMT-related markers in the indicated MEF2A-silenced, MEF2A-overexpressing, and corresponding control cells. (Student’s *t* test). Correlations between MEF2A expression and ZEB2 or β-catenin expression in xenograft tumors (**g**) and in 22 CRC patients (**h**). Representative IHC images are shown (right). Magnification: 100×. Scale bar: 100 µm. All experiments were repeated independently three times. The values are shown as the mean ± SD.

RNA sequencing was performed to identify the downstream targets and signaling pathways responsible for MEF2A OE-mediated malignancy-promoting phenotypes in CRC. The results showed that MEF2A OE results in upregulation of 75 genes and downregulation of 10 genes (|Log2FC|>2, *p* < 0.05) (Fig. 4a). Gene set enrichment analysis (GSEA) was used to determine general functional features of significantly altered gene sets (Supplementary Fig. S4a, Supplementary Table S1). The results showed that the genes induced by MEF2A OE were mainly enriched in the pathways that activate the EMT, such as adherens junction and WNT signaling (Fig. 4b). Moreover, GSEA of MEF2A positive-associated genes in the GSE17536 dataset confirmed that MEF2A was positively correlated with the EMT (Fig. 4c). Comparison with significantly upregulated genes in the microarray dataset with EMT-related gene sets in the AmiGO2 database [23] demonstrated that the mRNA levels of two EMT-related TFs (ZEB2 and CTNNB1) were upregulated by MEF2A OE in HCT116 cells (Fig. 4d). To validate the results, we knocked down or overexpressed MEF2A in CRC cells and detected the expression of mRNAs and proteins of ZEB2, CTNNB1, and other EMT-related markers. The results showed that upregulation of MEF2A increased the expression of *ZEB2* and *CTNNB1* and promoted the EMT, and downregulation of MEF2A achieved the opposite results (Fig. 4e, f). In xenograft tumors and CRC tissues, the expression of MEF2A, ZEB2, or β-catenin was highly positively correlated (Fig. 4g, h, Supplementary Fig. S4b). Thus, we selected *ZEB2* and *CTNNB1* as the candidate targets of MEF2A for subsequent experiments.

### MEF2A promoted the migration and invasion of CRC cells by directly targeting *ZEB2*

**Fig. 5:**
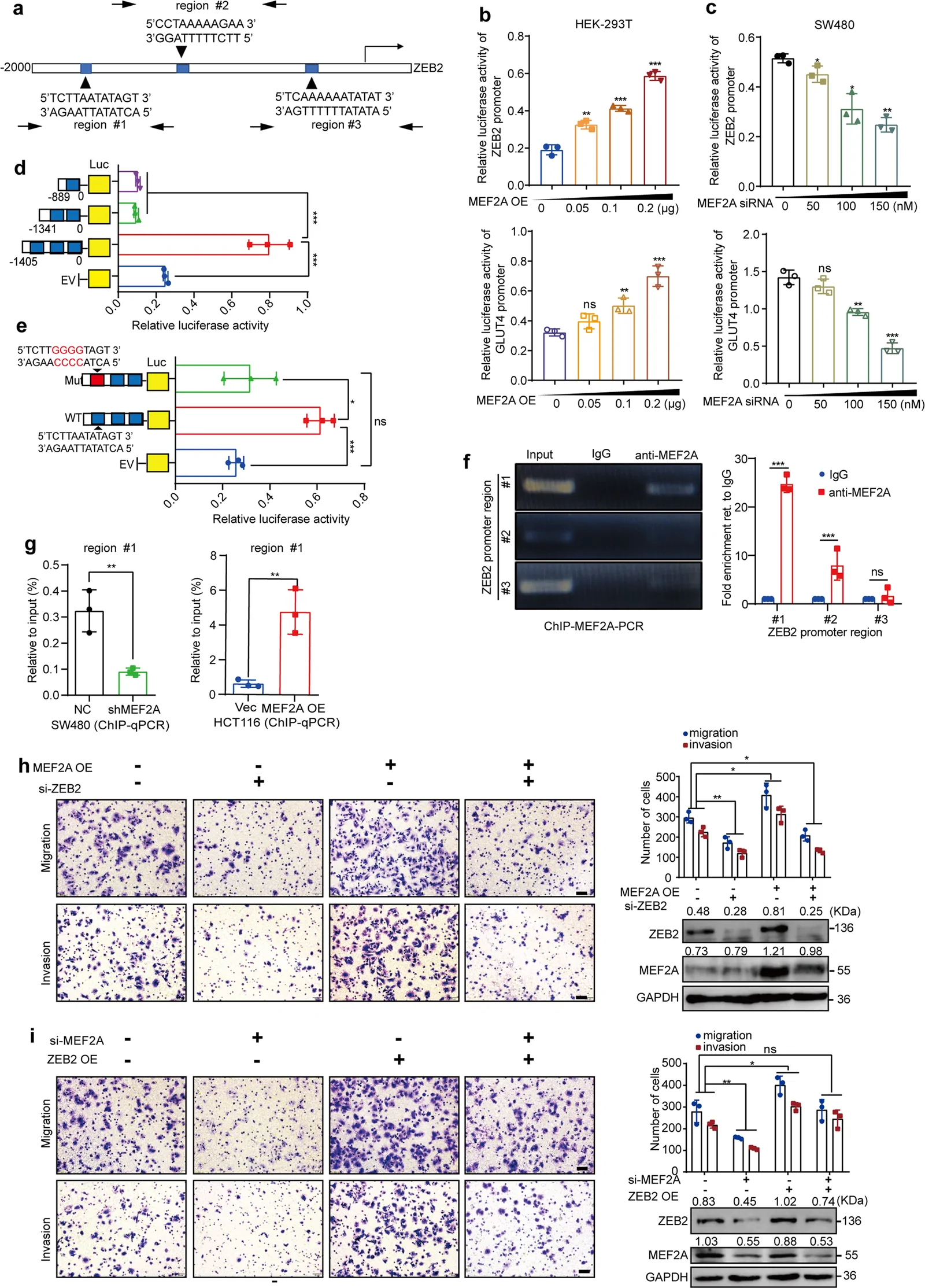
MEF2A promoted the migration and invasion of the cells by direct targeting of ZEB2. **a** Scheme of MEF2A-binding sites on the *ZEB2* promoter. Relative luciferase activity in HEK-293T and SW480 cells cotransfected with the *ZEB2* or *GLUT4* promoter and various concentrations of the *MEF2A* OE plasmid (**b**) and *MEF2A* siRNA (**c**). *GLUT4* promoter was used as a positive control (Student’s *t* test). Dual-luciferase reporter assay of MEF2A-overexpressing HEK-293T cells transfected with the truncated *ZEB2* promoter (**d**), WT *ZEB2* promoter, or mutant (Mut; AATA to GGGG) *ZEB2* promoter (**e**). WT, wild-type; EV, empty vector (Student’s *t* test). **f**, **g** ChIP-qPCR analysis of MEF2A binding to the *ZEB2* promoter. **h**, **i** Transwell assay was used to detect the rescue effect of ZEB2 on the migration and invasion. All experiments were repeated independently three times. The values are presented as the mean ± SD.

*ZEB2* is a potential target of MEF2A (Fig. 4d). Thus, we evaluated a possibility that MEF2A directly binds to the *ZEB2* promoter, which contains three putative MEF2A-binding regions with a relative profile score threshold of 85% (Fig. 5a). The results of the dual-luciferase reporter assays indicated that the luciferase activity driven by the *ZEB2* and *GLUT4* promoters was dramatically enhanced by MEF2A OE in a dose-dependent manner (Fig. 5b). The *GLUT4* promoter was used as a positive control. Consistently, knockdown of endogenous MEF2A levels resulted in a decrease in the promoter-driven luciferase activity (Fig. 5c).

The *ZEB2* promoter was truncated into three parts (−1405 to 0, −1341 to 0, and −889 to 0 bp according to predicted binding sites) to identify a specific binding site. The results suggested that the first region (−1405 to −1341bp) of the *ZEB2* promoter was required for MEF2A-dependent upregulation of luciferase activity (Fig. 5d). The sequence (TCTTAATATAGT) in the first region was considered to be the most likely binding motif responsible for *ZEB2* transactivation by MEF2A. Thus, we mutated the MEF2A-binding motif from AATA to GGGG, and the results indicated that MEF2A did not enhance the luciferase activity driven by the mutant ZEB2 promoter (Fig. 5e). Chromatin immunoprecipitation (ChIP) analysis also demonstrated that MEF2A was mainly bound to the *ZEB2* promoter at the −1351 to-1339 bp region (Fig. 5f) and that the binding was dramatically increased or decreased by MEF2A up- or downregulation, respectively (Fig. 5g). These results indicated that MEF2A directly promotes *ZEB2* transcription.

The MTS and Transwell assays SW480 cells with knockdown *ZEB2* by siRNA were used to investigate whether MEF2A affects CRC motility by promoting the expression of ZEB2 (Supplementary Fig. S5a, b). The results indicated that ZEB2 was not required for CRC proliferation (Supplementary Fig. S5c) but was important for cell migration and invasion (Supplementary Fig. S5d). As expected, impaired migration and invasion caused by ZEB2 downregulation were not rescued by overexpression of MEF2A (Fig. 5h). However, overexpression of ZEB2 in MEF2A-silenced cells effectively restored the migration and invasion abilities (Fig. 5i). Overall, these data suggested that MEF2A facilitates the migration and invasion of CRC cells by regulating the transcription of ZEB2.

### MEF2A directly upregulated *CTNNB1* and enhanced the activity of the WNT/β-catenin pathway

**Fig. 6:**
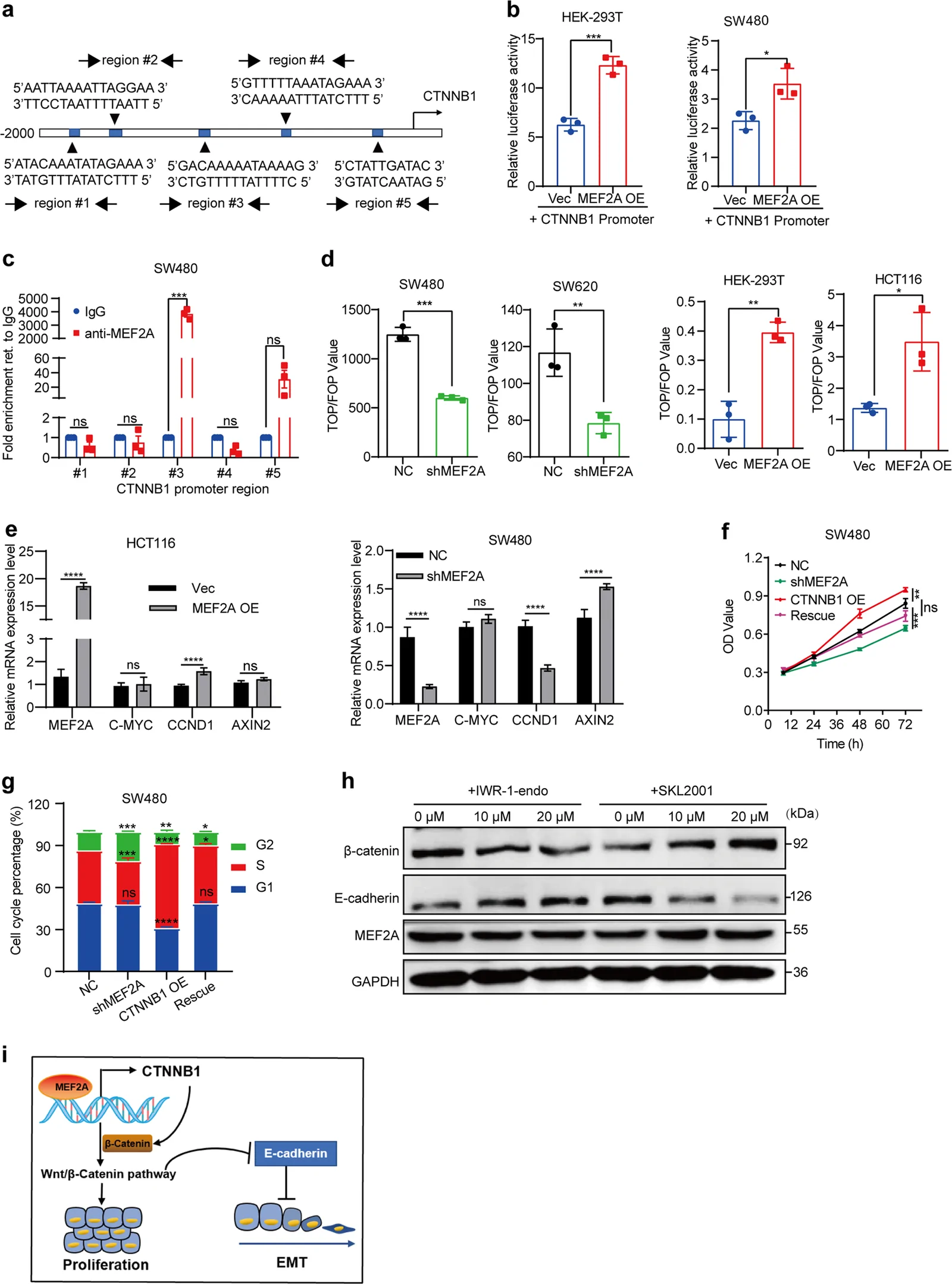
MEF2A promoted cell growth by regulating β-catenin. **a** Scheme of MEF2A-binding sites on the *CTNNB1* promoter. **b** Dual-luciferase reporter assay of the regulatory effect of MEF2A on *CTNNB1* promoter-driven luciferase activity in HEK-293T and SW480 cells (Student’s *t* test). **c** ChIP-qPCR detection of MEF2A-binding sites on the *CTNNB1* promoter (two-way ANOVA). **d** The TOP/FOP flash system was used to detect the effects of MEF2A on the activity of WNT signaling (Student’s *t* test). **e** The expression of mRNA of the target genes of the WNT/β-catenin pathway in HCT116 and SW480 cells (Student’s *t* test). MTS assay and flow cytometry were used to detect the rescue effect of β-catenin on cell proliferation (**f**) and cell cycle distribution (**g**) (Student’s *t* test). **h** Western blot analysis of the effects of a WNT signaling inhibitor (IWR-1-endo) and an activator (SKL2001) on EMT. **i** The working model of MEF2A-promoted proliferation of CRC cells. All experiments were repeated independently three times. The values are presented as the mean ± SD.

Analysis of the JASPAR database identified five potential MEF2A-binding sites in the promoter region of *CTNNB1* (−2000 to 0 bp) (Fig. 6a). Dual-luciferase reporter assays showed that MEF2A greatly enhanced *CTNNB1* promoter-driven luciferase activity in HEK-293T and SW480 cells (Fig. 6b). ChIP analysis revealed that region #3 (−1428 to −1452 bp) and region #5 (−929 to −943 bp) of the promoter were the main binding sites of MEF2A (Fig. 6c). β-Catenin is the central component of the Wnt/β-catenin signaling pathway. Since MEF2A increased the mRNA and protein levels of β-catenin via direct transcriptional regulation, we hypothesized that MEF2A is involved in the activation of the WNT/β-catenin pathway. TOP/FOP flash system was used to demonstrate that treatment with MEF2A siRNA or MEF2A-OE plasmid caused a severe reduction or an increase in β-catenin-dependent Tcf/LEF transcriptional activity, respectively, in HEK-293T and CRC cells (Fig. 6d). *CCND1* is the target gene downstream of the WNT/β-catenin pathway and a core protein of the cell cycle; the expression of *CCND1* was increase or decrease, respectively (Fig. 6e). Thus, we evaluated the role of β-catenin in cell proliferation induced by MEF2A. The results indicated that *CTNNB1* OE rescued the proliferation, which was decreased by MEF2A silencing (Fig. 6f, g). Treatment of CRC cells with a Wnt inhibitor (IWR-1-endo) or an activator (SKL2001) for 24 h indicated that suppression or activation, respectively, of Wnt signaling had a low magnitude effect on MEF2A expression but greatly increased or repressed, respectively, the expression of E-cadherin (Fig. 6h). The results suggested that a high level of MEF2A activates Wnt signaling by increasing the expression of β-catenin. On the other hand, MEF2A OE effectively promotes the EMT by activating the Wnt signaling pathway (Fig. 6i).

## Discussion

Despite many years of the investigations, only a few studies reported convincing evidence of the molecular mechanism of the regulation of the growth and metastasis of CRC by MEF2A. Understanding of the mechanisms of MEF2A-governed growth and metastasis of the tumors will assist with identification of potential therapeutic targets and will improve the survival rate of CRC patients by preventing CRC progression. The present study demonstrated that a high level of MEF2A in CRC tissues increased metastasis and decreased overall survival time, and the mechanism by which MEF2A drives the malignant phenotypes of CRC were preliminary characterized.

Implications of MEF2A in human cancer were discovered in prostate cancer [15], and MEF2A was shown to participate in stress-induced progression of prostate cancer as a p38 substrate. MEF2A is phosphorylated by p38MAPK in gastric cancer and promotes tumor proliferation and metastasis [13]. In breast cancer, MEF2A is activated by TGF-β and mediates TGF-β-induced breast cancer metastasis by upregulating MMP10 [24]. Additionally, in ovarian cancer, MEF2A was considered one of the transcription factors (TFs) responding to norepinephrine-involving tumorigenesis [25]. In the present study, MEF2A functioned as a TF by binding to the promoter region and initiating *ZEB2* transcription to promote metastasis. ZEB2 directly downregulates the expression of E-cadherin to drive the EMT [26]. High levels of ZEB2 are associated with metastasis of various cancers, including CRC [27]. Nuclear ZEB2 is associated with early recurrence in CRC, and some studies suggested that ZEB2 can be used as a molecular marker for risk stratification of patients with CRC [28].

Inactivated β-catenin is located in the cell membrane and cytoplasm and is involved in cell adhesion by forming a complex with E-cadherin [29]. The activation of canonical WNT signaling is characterized by the cytoplasmic stabilization and nuclear translocation of β-catenin [30]. Aberrant WNT signaling is critical for the maintenance of malignant CRC [31]. MEF2A has been reported to be a direct interaction partner of β-catenin in vascular smooth muscle cells (VSMCs) [32] and to impact the activity of the noncanonical WNT pathway via the CaKMII/HDAC4 axis in the maladaptive cardiac remodeling process;[33] however, the effects of MEF2A on the WNT signaling pathway in the tumors have not been extensively studied. The present study demonstrated that β-catenin is the target of MEF2A and overexpression of MEF2A substantially increased the activity of WNT/β-catenin signaling in CRC. On the other hand, the activation of WNT/β-catenin signaling may disrupt the formation of the β-catenin/E-cadherin complex, thereby reducing the adhesion between the cells and promoting the EMT.

MEF2A is a well-known transcription factor that regulates hundreds of genes. Interestingly, the data of a microarray testing obtained in the present study and analysis of the GSE17536 dataset indicated that several genes positively associated with the expression of MEF2A belong to the interferon (IFN) pathway. In *Drosophila*, *mef2* has been identified as a key transcriptional regulator of the innate immune response [34]. Activation of MEF2C by lipopolysaccharide (LPS) increases c-jun gene transcription involved in inflammatory regulation [35], and MEF2C is downregulated upon chronic exposure to IFN-β in microglia [36]. MEF2 interacts with IRF2BP2 (interferon regulatory factor 2 binding protein 2) in macrophages to promote the expression of KLF2 at transcriptional level and thus demonstrated an anti-inflammatory effect [37]. Moreover, MEF2D has been shown to transactivate the expression of PD-L1, which is mediated by the stimulation of IGN-γ [12]. However, the role of MEF2A in the immune response or interferon response was not extensively studied. The results of the present work provide insight into the function of MEF2A in immune regulation.

MEF2A transcriptional activity is regulated by associations with several transcriptional cofactors and by posttranslational modifications (PTMs). The transcription activity of MEF2A is always enhanced by phosphorylation by p38MAPK at Thr312 and Thr319 [38]. Phosphorylation of Ser408 of MEF2A induces sumoylation of Lys403, thus repressing the transcriptional activity of MEF2A [39]. Class II HDACs are important corepressors of MEF2A that bind to MEF2 to suppress its functions [40]. In addition to transcriptional activation and repression, phosphorylation plays a critical role in MEF2A degradation. Phosphorylation by Cdk5 and a calcium-sensitive kinase is sufficient to promote the degradation of MEF2A by caspase-dependent cleavage [41]. Caspase-3/7 activation is a direct pathway for MEF2A degradation [42]. The ubiquitin-proteasome and autophagy-lysosome pathways are two other pathways for MEF2A degradation. During oxidative stress, MEF2A can be physiologically degraded via the autophagy-lysosome pathway mediated by Hsp8 [43]. Ubiquitination of MEF2A promotes its degradation via the ubiquitin-proteasome pathway in neurons [44]. The degradation pathway of MEF2A in neurons was extensively characterized; however, only a few studies reported extensive degradation and posttranslational modifications of MEF2A in tumors.

**Fig. 7:**
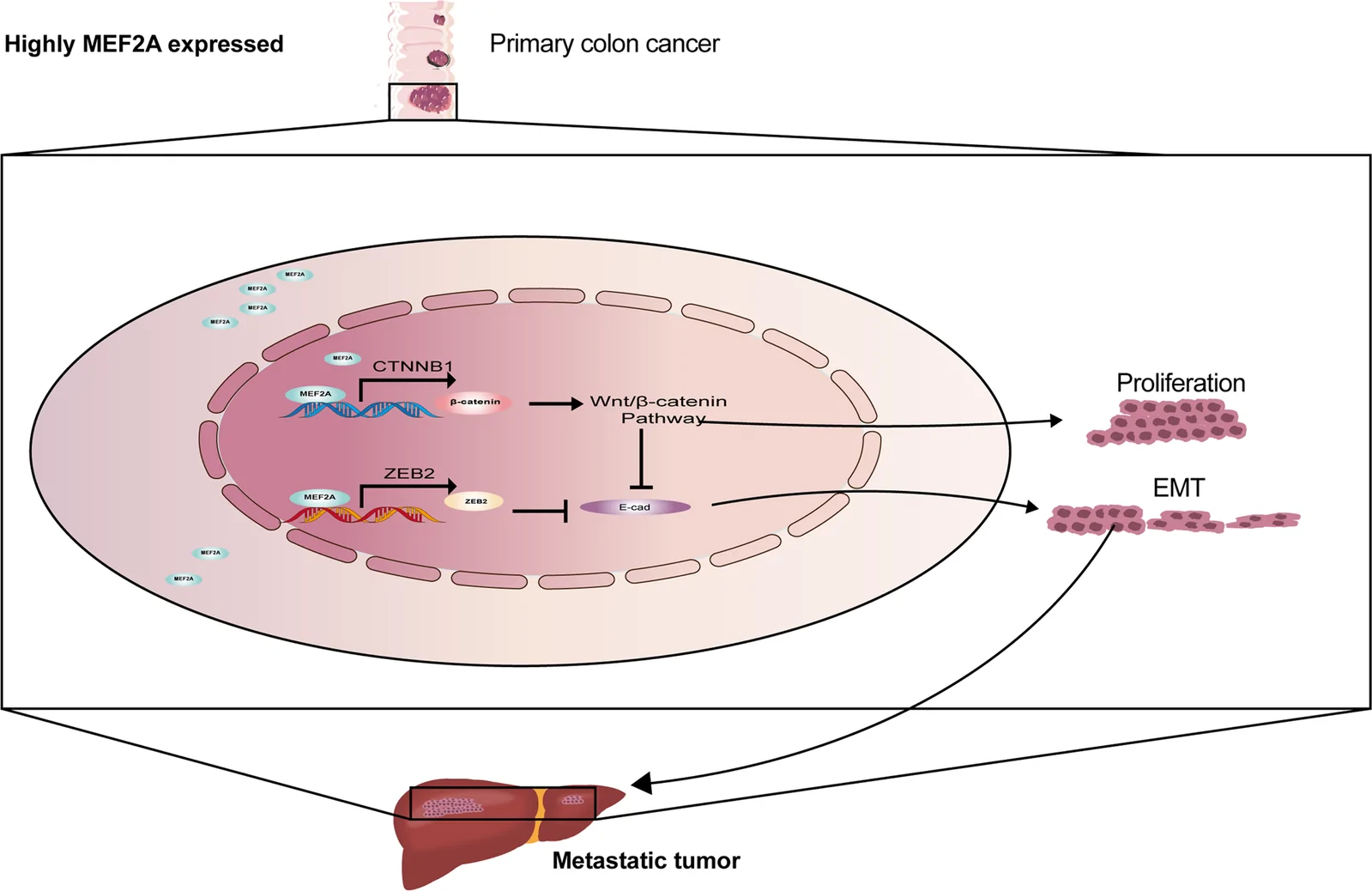
A model of MEF2A functions. Schematic description of the main molecular mechanism involved in the dual role of MEF2A in the regulation of CRC proliferation and metastasis.

In conclusion, MEF2A promoted the proliferation and metastasis of CRC by activating the Wnt signaling pathway and EMT in a manner mediated by β-catenin and ZEB2 (Fig. 7).

## Materials and methods

### Animal experiments

The protocols for animal care and euthanasia were approved by the Institutional Animal Care and Use Committee of Central South University (Changsha, China). Five-week-old male BALB/c (nu/nu) nude mice were used in the experiments performed according to the approved protocols. For subcutaneous xenograft generation, the mice were randomly divided into four groups and subcutaneously injected with 1 × 10^6^ SW480-KD MEF2A, SW480-NC, HCT116-OE MEF2A, or HCT116-vec cells. Tumor volume (length × width^2^ × 0.5) was measured every two days. After 30–60 days, the mice were sacrificed, and the tumor nodules were collected for hematoxylin-eosin (HE) and immunohistochemistry (IHC) staining.

Two metastasis models were generated: (1) intraperitoneal injection with 3 × 10^6^ CRC cells (with stable MEF2A KD or OE) and (2) injection with 3 × 10^6^ CRC cells (with stable MEF2A KD or OE) via the tail vein. Each group included 5 mice. Metastasis was detected after 2 months. Organs were isolated for histopathological examination of CRC tumors. All animals were included in the analysis, and investigators were not blinded during data acquisition and analysis.

### Human tissue samples

All CRC specimens were acquired from Xiangya Hospital, Central South University (Changsha, China). The present study was approved by the Protection of Human Subjects Committee of Xiangya Hospital. Informed consent was obtained from each patient. A total of 161 pairs of paraffin tissue samples from patients diagnosed with CRC between 2011 and 2013, 55 freshly removed cancer tissue samples, and 42 adjacent noncancerous colonic tissue samples obtained by surgical resection were used in the present study.

### Statistics

The results are shown as the mean ± SD or SEM. The sample size was determined by statistical analysis of variance during experiment planning. Statistical significance was determined using GraphPad Prism 8.0 and SPSS software by *t*-test, one-way ANOVA, or log-rank test. Statistical significance was defined as *P* < 0.05 (*P < 0.05, **P < 0.01, ***P < 0.001).

Detailed information about the materials and methods used in the present study is available in Supplementary materials and methods.

## Supplementary information

Supplementary Materials and methods

Supplementary figure legends

Figure S1

Figure S2

Figure S3

Figure S4

Figure S5

Table S1
