# Supplementary material for: MEF2A transcriptionally upregulates the expression of ZEB2 and CTNNB1 in colorectal cancer to promote tumor progression
Source: Oncogene. 2021 Apr 16;40(19):3364–77. doi: 10.1038/s41388-021-01774-w (PMC8116210; doi:10.1038/s41388-021-01774-w)
Supplement: Supplementary file 1 — Supplementary Materials and methods [file 41388_2021_1774_MOESM1_ESM.doc]

**Supplementary materials and methods**

**Cell culture**

The human CRC cell lines HCT116, HT29, SW480, and SW620 and the human embryonic kidney cell line HEK-293T were purchased from American Type Culture Collection (ATCC). The normal colon epithelial cell line NCM460 was obtained from the Cell Bank of the Chinese Academy of Science (Shanghai, China). All cells were maintained in RPMI-1640 (BioInd, Beit Haemek, Israel) containing 10% fetal bovine serum (BioInd) in an atmosphere of 5% CO_2_ at 37 °C.

**Plasmid, siRNA, and stable cell line construction and transfection**

Plasmids encoding human *MEF2A* and *CTNNB1* were produced by PCR amplification and subcloned into the pEGFP-C1/pCDH-CMV-MCS-EF1-Puro and pcDNA6 expression vectors, respectively. A *ZEB2* OE plasmid and *MEF2A* shRNA plasmid were purchased from Genechem (Shanghai, China). Plasmids expressing the *ZEB2* promoter (-1405 to 0, -1341 to 0, -889 to 0 bp), *CTNNB1* promoter (-2000 to 0 bp), and GLUT4 promoter (-2000 to 0 bp) were produced by PCR amplification and subcloned into the pGL3-basic expression vector. A mutant *ZEB2* promoter was constructed based on the WT promoter using a mutation kit (Vazyme, Nanjing, China). All sgRNA sequences with minimal off-target scores were chosen and subcloned into the lentiCRISPR V2 retroviral vector. All siRNAs were synthesized by RiboBio (Guangzhou, China) and used at a final concentration of 5 nM. Transfection was conducted with a jetPRIME kit (Polyplus Transfection, Illkirch, France) according to the manufacturer’s instructions. HCT116 cells were transfected with pEGFP-MEF2A or empty vector, selected with 800 μg/ml G418 (Sigma-Aldrich, St. Louis, MO) for one week and maintained with 400 μg/ml G418. SW480 and SW620 cells transfected with *MEF2A* shRNA or sgRNA were selected with 1 μg/ml puromycin dihydrochloride (MedChemExpress, Monmouth Junction, NJ). All stable cell lines were confirmed by qPCR and western blotting. The siRNA, shRNA, and sgRNA sequences are provided in the flowing table.

**siRNA sequences**

| Gene | siRNA sequences |
| --- | --- |
| MEF2A #1 | GCAGCCAGCTCAACGTTAA |
| MEF2A #2 | GTGGTAATCTTGGAATGAA |
| MEF2A #3 | GCCCTTCTGTAAAGCGAAT |
| ZEB2 #1 | CTGCAAGGCTGAAGAAATT |
| ZEB2 #2 | GCATGTATGCATGTGACTT |
| ZEB2 #3 | GAACAGACAGGCTTACTTA |
| CTNNB1 #1 | TAAGCCGGCTATTGTAGAA |
| CTNNB1 #2 | GCCACAAGATTACAAGAAA |
| CTNNB1 #3 | GCTGAAACATGCAGTTGTA |

**shRNA sequences**

| Gene | shRNA sequences |
| --- | --- |
| MEF2A | GTGGTAATCTTGGAATGAA |

**sgRNA sequences**

| Gene | sgRNA sequences |
| --- | --- |
| MEF2A #1 | GCTGAGTACACAAGTCCTTG |
| MEF2A #2 | GCTGAGTACACAAGTCCTTG |
| MEF2A #3 | GACACGACTGGGGTAGCAAG |
| MEF2A #4 | ACAGAGCACACTAAGTTCAT |

**Promoter sequence**

>FP004007 ZEB2_1 :+U EU:NC; range -2000 to 0.
GTTAATTTATCCAGCAGCTATTTTCATTATAATAAATACTTAAATTAGGAAAGAGGTGAG
TAACCAACTGTGAAAATCTACTTTCTTGCATCCTAGAACAAGATCTAGCTGTGGTCGTGC
TAGGTAGCTATTAACCACCCCCACCCCACTCCCCCGCCCCGCAAAAAAAAATACAAAATA
CAAAGACGTATACCTCTGGGAGTGTGGATGTGGCAACCCATGTGGTGGCTTGTCCCTCCT
TTGAGTGCAATTGCCAGTGACTCTTGCATTCAAAGTCTTGCTTTAGGCACACATTCAAAG
CACAGTTCAAAGCAGTTTATCTTAATATAGTGTTGGATCAGGGTATTATTTGTTTCAGAT
TTCATCAGACTGGAAACAGGAGGGTGTAACATATTTAACAATATTAAAGCTTAAAAATTG
GTGTGAGGCAGCTCTCTGGTCCTCTTAACACCTCCACCTTTACCTTTCCGAAGAATCCTA
AATGCTGTGAGGCTAGAAGTTTATTTGTGCTCTGAGGTTCTCAAGCACTTTCACTCTTTA
GGAAAAAAAAAAAAGTCTCACAGTTATTTAAGAAATTGAGCCTGAACCATCCGAGGATAG
GAGATTTCTTCCTTTTCCGATAAATGCCTGTGCTCAGCATCCTCAAATTTGGCTGTCTTT
ATTTACTTCTATATTAGTTCCAATTATGTGCAGTGTAAACCAGCTTTTTGATTAACAAAA
CAGCAGAGCATTGGTTAAGAACACTTAAACTTCAAAAAAATTCCCCTGGGAAGTGGGCTC
TGAATTGGTTAATAATGTGCAGTACTTGCTAAATTGCTGACTTCCAGATGTGGAAAATAT
CTTTCTTGTTTAGGACCTATGTAACCTGATTGGATTACGACAGAAGCGTCACGTTGGAAG
CTTTTGAGTGATTATTTAATTAACCATACAGTAGAAAGCTGTATTCTCCAGGAAATCCCT
TCCATATTTGCATAACCAATCCCTTCAGAGCAAAGGTGGAGTCTTTTCTTTTTAATCTTA
CTTTAATTGTAATATCAAAAAATATATACTCCAAAACTTAGACCCCATGCCGTTTAATAT
CATGCTCTCCCTTCCCTGCTAAGTTTCTCTATGGCCTTTCTCGTTTCTCCTCCTGCCTCC
CTACACACTCTCCCTTACTTTGTAGTGAGGTCTCCCCGAGGTGTAGAGAGATTCAGAGAT
CGGCCAACCGAGTGTTCTATTTTAATTTACTTAGAGACCCTTTATTAAAATGCCAAACTA
CTTTTTAATATTGGGATCCAGTCCAGAAATTCATCATGCACACACCCTAATACACATGCC
CTAAGATGCAGCTCCCATGCAGCATTTTTTTTTCTGGCTCTGGTACCTAAAAAGAAAAAA
ATAACAATAAGAGAAAGGGCAGAGAACTTTGTTCCAGAAGCTGTACTGAGATACCTACAC
AATTTGATGTGCATCTCAAATCTGGTCATTAGAGATATCTGTATAAGAAGAGACTATCTG
GATTGAGGACCCGGGATCTTTCCCTTTAACTTTCGCCCCTTGGAGTTCTCCAGTTCTGTG
AATGGTGTGCACCGTTTTCCGCCCTGTACTCTGTAGGATTTAGTGATGAGGATAATGATG
CCAAAGGCTTGACGGGCGGGGAGGGGGGGGTGGAGGGGGGGAGAAGGGAGGGAGGGGGGA
GGGAGGCGAAGGCGAAAGGGAGGGAGAGGAGGAAGGGAGGGAGGTGGAATTTCATTTCTT
CCACTAAAGCGTTTGCGGAGACTTCAAGGTATAATCTATCCCAGATCCTTTCCCAGAGAG
AAACTTGGCGATCACGTTTTCACATGATGCTCACGCTCAGGGCGCTTCAATTATCCCTCC
CCACAAAGATAGGTGGCGCGTGTTTCAGGGTCTCTCGTCTCTCTCCTACAGAAAAGAAAA
AGAAAAAAATGTCATTAGAAGAGGCGTAACACGTCAGTCCGTCCCCAGGTTTGTGTTTCC
TGGAGTGGCCGAAAGAGATCA

>FP022586 SLC2A4_1 :+U EU:NC; range -2000 to 0.
CGGATTTTTTTTTTTCTTCTTGAGACGGAGTCACTCTGTCGCCAGGCTGGAGTGCAAGGG
CACGATCTTGGCTCACTACAACCTCCACCTCCTGGGTTCAAGCCATTTTCCTGCCTCAGC
CTCCCGAGTAGCTGGGATTACAGGTGTGCATAACCACGCCCGGCTAATTTTTGTATCTTT
AGCAGACATGGGGTTTCTCTATGTTGGCCAGGCTGGTTTCAAACTCCTGACCTCAGTCGA
TCCACCTGCCTTGGCCTCCTAAAGTGCTGGGATTACAGGCATGAGCCACCAGGCCGGGCC
GGCATTCCAGATTTTTCAGGGGATTAGTGCAGCAAAGGAATCAAGAAGGGATGTAAAGGC
ACAGTGTGTTCTGGGTACAATAAGGACTTAGGCATTGCCCAGAACAGGAGGCGAAGGAGA
TAGAAGGAGAGGCAGGAGAGATAGGTAAGGCCAGAGATCGGATAAGAGAGGCAGGAGGTT
TTGTTCACTCTGAAAAGGGATTTGAACTTGGCAATTGGGGCAACAGAGACAGTGACTTCT
TGCTTGAGAGATGAGATTGGACCTTCGAAAATTGTTCTCTGCCCTCGTCATAAAGGAAAT
AAGAGGAGCACGAAGACCAGTGAGGGTGATGGTGATCTGGACTGAAGTGGCAGCCGCCAC
GGAGAATATCGGATGAATGTGAGAGAGTTTTGGAGGTCAAAGCACCAATGTTGGAAACTA
ACTGGATAAACGAGGAGAGCGGCGCAGGACAGGAGGAATCGAGCCTGACTTCTACCATAG
GGGTGACTGGGCGGGTAATTCATTGAAATAAGGAAGTTAGGAGGAGGAGCAGGTTTGGAC
ATGCTGATCACTAGAGCTGCCACATCCGGGCGGTAACGAACACCTGGATCTGCAGCTCCA
GAGAAGGGCCTGGGTCAGATGTCACTGAAGCCCTATGGTGGCGGAAAGGCGAGAAATAGT
GGGTTGAGATTCCAAGTGCAATCCACTGCGGCTCCTCGCTCGCCCTCCAGGTGGCAGCAC
AACCCTGCGCTTCCGAAGCCCGTTTTCTGAGCCAGACACTCTCCACGCTCTGGGTATTTC
GGCTTCTCTCTCCCCACACGCCGACCCTAGGTCGCGCACTTTCTGCCTGGCAGAATTTGG
CCGAGGATCCAAACCCGGAGCAGCCTCCAGAGAGCGTGTCGTTCACGCGGCCAGCATATG
CTCAGAGACCTCAGAGGCTCAGAGACCTCAGGGCTGGTGGTGTGGTCGGTTGTGACCACT
TGTCCCTCGGACCGGCTCCAGGAACCAACCTGGGGAATGTGTGTAGGGGAAGGGCGGGAT
AGACAGTGCCCGGAGCAGGGAGGCGCTGAAAGACAGGACCAAGCAGCCCGGCCACCAGAC
CCGTTGTGGGAACGGAATTTCCTGGCCCCCAGGGCCACACTCGCGTGGGAAGCATGTCGC
GGACTCTTTAAGGCGTCATCTCCCTGTCTCTCCGCCCCCGCCTGGGACAGGCCGGGACGC
CCGGGACCTGACATTTGGAGGCTCCCAACGTGGGAGCTAAAAATAGCAGCCCCGGGTTAC
TTTGGGGCATTGCTCCTCTCCCAACCCGCGCGCCGGCTCGCGAGCCGTCTCAGGCCGCTG
GAGTTTCCCCGGGGCAAGTACACCTGGCCCGTCCTCTCCTCTCAGACCCCACTGTCCAGA
CCCGCAGAGTTTAAGATGCTTCTGCAGCCCGGGATCCTAGCTGGTGGGCGGAGTCCTAAC
ACGTGGGTGGGCGGGGCCTTTTGTTCCAGGGACTCTTTTCTCAAAACTTCCCAGTCGGAG
GCTGGCGGGAACCCGAGAGGCGTGTCTCGCCAGCCACGCGGAGGGGCGTGGCCTCATTGG
CCCGCCCCACCAACTCCAGCCAAACTCTAAACCCCAGGCGGAGGGGGCGTGGCCTTCTGG
GGTGTGCGGGCTCCTGGCCAATGGGTGCTGTGAAGGGCGTGGCCCGCGGGGGCAGGAGCG
AGGTGGCGGGGGCTTCTCGCG

>FP005181 CTNNB1_1 :+U EU:NC; range -2000 to 0.
AATGATTCCATTATTTTGGTAATGGAACAGAGAAATACAAATATAGAAACGTTTTTATCC
TTTAGATCTTCTAATGGTGCAGATATTTCTTAACCAATTTCAAGAGTGCCTTATTCAAAA
ACAAAAAACCTGCACATAGAAAAAGCAAACATGTTTTTTTAAAAAAAGCCCTGTAAAAGT
GGTTAATCTGTCGTCTGATTTTCAATGTATGACTTTAATCAATTGCATATTCCTTAATTC
TGCAAATGACAATTAAAATTAGGAATCAGTACCTGAAAACGCCATTTATACTTTTGAGTT
TATATTAGAAACACGGTTTTGATGAAATACCTTTTTCGGTTCAATCTTTTTGAATTGTGA
CCACAACCAATAGCTACACTTAGATTCTACAGAATTAGTAGGAAAGATACAAATGAGAAG
GTCTTTCTAGACAATGGATCTCACTGAACATCATCTTAGTGTGTAGACTTTTTCCACTGC
AGTGGCCCACATGTGCTATGGTGACAGACGGCAGTTGGCATTACCACTTATATAGTAAAA
TATCTTTAAGACAAAAATAAAAGTTATTTAAAGAATACGCTGGCCCTGAAACATGAGCTG
TGCCTTAAACTACATATCCTACCTCCATGAGTGAATGAATAAGTGGGTTTTCCGCCTGCA
TCTATGTGTTAAGACCTATTGTTTGCTTGTATTAATTGTAGTCCTGGGATAAAAGCACAG
AGGTAACTTTCACTGCTGCTTTTTGTACTCTCTCCAATGTTTTGGAGGAAAAATAAGCAC
AAACAATAGCCTAGAGAAACTGAATCGATCATACTTGTTGCAGCTTCGACAAACGTCAAT
TTTGCTGCATTAGAATGGGAAACATTTTTCAGTCTATTGAAATGAATTACAAACGTTTTT
AAATAGAAAATTAGTTAAAAAATTGGAGGCTGCTTAATCGATAGCTTTCTCTATAAACAT
ACTTGGATTTCACAAATAAGTAATACCGTAAAAATCTTCTTCTCCAAAGAAAAATCCCCA
CAAATAAATCTATTGATACCTAGTGACAAGTGGAACCAGATAAAAATGGAATCTATAAGA
ATTAACCTAATTGACAGCGCTCTGGAGCTAATCCATTTCCATTAGTTATTTGTTCACAGT
AGGTACTCCTAAGGACTTGTTGAATTGCGGGCTTGGCGCCCGTTCTACGGAGAGTTCACA
GCCTTCGTGAGTGGGGACAGAAGGCGGCTCGGCCCGGTGATTCAGGTCGAAATTCAAGCT
GAACAGCCTGCTGAGAGGTGGGATCCACCATCCGGACAGTGGGGGGCTTTGGGGGTGCTG
TGAGACTGGGCTGCGACCCAGGTCCAGCAGGGAGTGTGCGGCACAGACCACAAGGTCGGC
GAGGCCCCCTAACCCGCGCCCGGCCGGGAACCCGCAGACCAGCGACGGGGCAGCTGCGGG
GCCAGGAGCGCCCCAAGACGGGCGGGCGCTGAACCCGAGCCCCTGCCGCCGCCCTGGCCC
CGAACTTCCGCCCTCCCAGGACCTGTCCCGGCCGCCCCGAGCGGTACTCGAAGGCCGGGG
CCGAGATGCCACCTTCCGCAGGCCGCGGGAAAGGCGCGCCGAGTCCTGCAGCTGCTCTCC
CGGTTCGGGAAACGCGCGGGGCGGGGGCGTCGGGCTTGGGACAGGGGAGGATACCAGGGC
CACCTTCCCCAACCCAGGCCGCGGGGGCCCGGCCTCCCCGATGCAGACCACAGCGCCCTC
ACGGGCTGCCCTCAGGCCGCGCAGCGGGCAGCCGCCAGCCGTCACCCCGGGGAGCGTCCG
TGGGGTGCCCAGGCACCCCACCCCGGCCCGGGGCGCTCAGACGGCAGCAGACTGCTGGGC
GGCGCGGGGACTACTTTCCACCGCCCCCTCGCGCCCCGCCCCTTGTCCTCGCGCGGCGGA
ACGCTCCGCGCTGCGCCGGTGGCGGCAGGATACAGCGGCTTCTGCGCGACTTATAAGAGC
TCCTTGTGCGGCGCCATTTTA

**Real-time quantitative PCR**

Total RNA was extracted using TRIzol reagent (Invitrogen, Thermo Fisher Scientific, Waltham, MA), and RNA of proper integrity was reverse transcribed into cDNA using the GoScript Reverse Transcription System (Promega, Madison, WI). Real-time quantitative PCR was performed according to previously described methods(1). The relative expression level was calculated by the 2^−ΔΔCt^ method. The results were normalized to GAPDH expression. The sequences of all the primer sets used in this study are listed in below.

**qPCR primers sequences**

| Gene | Primer sequence |
| --- | --- |
| MEF2A-QF | GATGCCTCCACTAAATACCC |
| MEF2A-QR | ACTGCCCTCCAGCAACAAGA |
| MEF2B-QF | GGAAGTTCGGGCTGATGAAGAAGG |
| MEF2B-QR | GCATACTGGAAGAGGCGGTTGG |
| MEF2C-QF | GTATGTCTCCTGGTGTAACACA |
| MEF2C-QR | TGTTCAAGTTACCAGGTGAGAC |
| MEF2D-QF | GGCTGTCGCTAGGCAATGTCAC |
| MEF2D-QR | CTGTGGCTGTGGCTGCTGTG |
| CDH1-QF | ACCAGAATAAAGACCAAGTGACC |
| CDH1-QR | CCTCCAAGAATCCCCAGAATG |
| CDH2-QF | CGATAAGGATCAACCCCATACA |
| CDH2-QR | TTCAAAGTCGATTGGTTTGACC |
| VIM-QF | TGAATGACCGCTTCGCCAACTAC |
| VIM-QR | CTCCCGCATCTCCTCCTCGTAG |
| ACTA2-QF | TCGTGCTGGACTCTGGAGATGG |
| ACTA2-QR | CCACGCTCAGTCAGGATCTTCATG |
| ZEB2-QF | AAGAGAACTTTTCCTGCCCTC |
| ZEB2-QR | ATTTGAACTTGCGATTACCTGC |
| CTNNB1-QF | GTTCAGTTGCTTGTTCGTGC |
| CTNNB1-QR | GTTGTGAACATCCCGAGCTAG |
| CCND1-QF | GTCCTACTTCAAATGTGTGCAG |
| CCND1-QR | GGGATGGTCTCCTTCATCTTAG |
| C-MYC-QF | CGACGAGACCTTCATCAAAAAC |
| C-MYC-QR | CTTCTCTGAGACGAGCTTGG |
| AXIN2-QF | CTCCGAGCTCACACTCAATTC |
| AXIN-QR | GACAGGTGATCGTCCAGTATC |

**Cell proliferation, invasion, and migration assays**

For the cell proliferation assay, cells were seeded in 96-well culture plates (5,000 cells/well). At the indicated time points, the cells were treated with 10 µL MTS (Promega) for 2 hours, and the absorbance was detected at 450 nm. The Transwell assay was performed as described previously(2). The results were calculated with ImageJ (National Institutes of Health, USA).

**Luciferase reporter assay**

HEK-293 cells were seeded in a 24-well plate at 40-50% confluency, and cotransfected with *MEF2A* siRNA or MEF2A OE plasmid along with the *ZEB2/GLUT4/-CTNNB1* promoter. After 48 hours, the luciferase assay was performed using the Dual-Glo Luciferase Kit (Promega). Firefly luciferase activity in each sample was calculated by normalization to Renilla activity.

**Cell cycle analysis**

For cell cycle analysis, the cells were transfected with the shRNA or OE plasmid after starvation for 24 hours and cultured for 48hours. The cells were collected, fixed in with ice-cold 70% ethanol at 4 °C overnight, and kept at -20 °C until analysis. The cells were stained with PI/RNase Staining Buffer (BD Biosciences, NJ, USA) and evaluated by a FACSCalibur system (BD Biosciences), following the manufacturer’s instructions.

**ChIP assay**

We used OE or shRNA plasmids to specifically up/downregulate MEF2A in CRC cells. A ChIP assay was performed by using a kit (Santa Cruz, Santa Cruz, CA) according to the protocol provided by the manufacturer. An MEF2A antibody (Santa Cruz, sc-17785) was used for ChIP. qPCR analysis was performed to detect the DNA fragments that bound to MEF2A. The sequences of the primers are listed in below.

**ChIP-qPCR primers sequences**

| Gene | Primer sequence |
| --- | --- |
| ZEB2-1F | GCATTCAAAGTCTTGCTTTAGGC |
| ZEB2-1R | GTTTCCAGTCTGATGAAATCTGAAAC |
| ZEB2-2F | AGAGCAAAGGTGGAGTCTTTTC |
| ZEB2-2R | GAGAGCATGATATTAAACGGCAT |
| ZEB2-3F | TAAGATGCAGCTCCCATGCA |
| ZEB2-3R | TGACCAGATTTGAGATGCACATC |
| CTNNB1-1F | AATGATTCCATTATTTTGGTAATG |
| CTNNB1-1R | CATGTTTGCTTTTTCTATGTGCAG |
| CTNNB1-2F | ACCTGCACATAGAAAAAGCAAACA |
| CTNNB1-2R | AGTATAAATGGCGTTTTCAGGTACT |
| CTNNB1-3F | AGTGGCCCACATGTGCTATG |
| CTNNB1-3R | AGCTCATGTTTCAGGGCCAG |
| CTNNB1-4F | TGTTGCAGCTTCGACAAACG |
| CTNNB1-4R | AGCTATCGATTAAGCAGCCTCC |
| CTNNB1-5F | GGAGGCTGCTTAATCGATAGCT |
| CTNNB1-5R | GCTCCAGAGCGCTGTCAATT |

**Immunohistochemistry (IHC)**

IHC was performed as previously described(4). Visualization reagent was used to visualize the results. Tissue staining intensity and the percentage of stained cells were scored. MEF2A staining were evaluated at high (400×) magnification in five fields. The intensity was scored as 0 (negative), 1 (weak), 2 (moderate), or 3 (strong); the percentage of stained cells was scored as 1 (0-25%), 2 (26-50%), 3 (51-75%), or 4 (> 75%), and comprehensive scores were calculated as staining   percentage × intensity. Finally, the expression level of MEF2A was defined as low or high according to the comprehensive score (cutoff value = 6).

**Bioinformatics analysis**

Xena UCSC website ([http://xena.ucsc.edu/#overview](http://xena.ucsc.edu/#overview)) was used to analysis the correlation between MEF2s expression and overall survival of patients in the TCGA Colon and Rectal Cancer (COADREAD) dataset. Microarray data from GSE17536 dataset of CRC specimens were downloaded from the GEO database. For Kaplan-Meier survival analysis, survival data including sample type (primary tumors), days to first biochemical recurrence and days to last follow-up were considered. The samples were divided into two groups, the high and low expression groups, according to the expression level of *MEF2s* using the Cox proportional hazards regression model in R. Next, we performed a 150-month survival analysis of MEF2susing Kaplan-Meier survival analysis by employing the survival package, and statistical significance was computed using the log-rank test. Molecular signatures that were enriched upon *MEF2A* expression were analyzed using GSEA.

**References:**

1. Chen Y, Zhang B, Bao L, Jin L, Yang M, Peng Y, Kumar A, Wang JE, Wang C, Zou X, Xing C, Wang Y, Luo W. ZMYND8 acetylation mediates HIF-dependent breast cancer progression and metastasis. Journal of Clinical Investigation 2018;128(5):1937-55.

2. Chen C, Xu ZQ, Zong YP, Ou BC, Shen XH, Feng H, Zheng MH, Zhao JK, Lu AG. CXCL5 induces tumor angiogenesis via enhancing the expression of FOXD1 mediated by the AKT/NF-kappaB pathway in colorectal cancer. Cell Death & Disease 2019;10(3):178.

3. Lu L, Lee Y, Chang C, Shun C, Fang C, Shao Y, Liu T, Cheng A, Hsu C. Increased Expression of Programmed Death-Ligand 1 in Infiltrating Immune Cells in Hepatocellular Carcinoma Tissues after Sorafenib Treatment. Liver Cancer 2019;8(2):110-20.
