# Supplementary material for: MEF2A transcriptionally upregulates the expression of ZEB2 and CTNNB1 in colorectal cancer to promote tumor progression
Source: Oncogene. 2021 Apr 16;40(19):3364–77. doi: 10.1038/s41388-021-01774-w (PMC8116210; doi:10.1038/s41388-021-01774-w)
Supplement: Supplementary file 2 — Supplementary figure legends [file 41388_2021_1774_MOESM2_ESM.doc]

**Supplementary figure legends**

**Figure S1.** **MEF2A was upregulated in CRC tissues and correlated with shorter survival time. (S1a)** The correlation of MEF2s expression and overall survival (OS) of CRC patients in TCGA Colon and Rectal cancer dataset. **(S1b)** The mRNA level of MEF2C and MEF2D in GSE17536 dataset. **(S1c)** Kaplan- Meier curve showing the influence of MEF2C and MEF2D on OS of CRC patients in GSE17536. **(S1d)** Relative mRNA expression levels of MEF2A and MEF2C in fresh CRC tissues compared with corresponding nontumor tissues (Student’s t test). The experiments were independently repeated three times. *P< 0.05, ***P < 0.001.

**Figure S2.** **The effects of down or upregulating MEF2A on cell proliferation and motility. (S2a)** Flow cytometry analysis of apoptosis after knocking down MEF2A in SW480 cells. **(S2b)** MEF2A silencing efficiency in SW480 and SW620 cells. **(S2c-e)** The MTS assay, flow cytometry and the Transwell assay were used to measure the cell growth rate **(S2c)**, cell cycle distribution **(S2d)**, and cell motility **(S2e)** after MEF2A was successfully silenced in SW480 and SW620 cells. **(S2f)** MEF2A OE efficiency in SW480 cells. **(S2g-i)** The MTS assay, flow cytometry and the Transwell assay were performed to examine the cell growth rate **(S2g)**, cell cycle distribution **(S2h)**, and cell motility **(S2i)** after MEF2A was overexpressed in SW480 cells. **(S2j-k)** MTS assay and Transwell assay showing the change of cell proliferation and migration after silencing MEF2C alone **(S2j)** or silencing MEF2C and overexpressing MEF2A simultaneously in SW480 cells **(S2k).** The experiments were repeated independently three times. The values are the mean ± SD, *P < 0.05, **P < 0.01; ***P < 0.001. All data in this figure were analyzed by Student’s *t* test.

**Figure S3.** **Metastatic lesions in the mesentery and diaphragm after MEF2A knockdown or OE. (S3a-d)** Formation of metastases after intraperitoneal injection of 3×10^6^ CRC cells (n=5). n/5 indicates that there were 5 mice in each group, and n represents the number of mice that formed metastatic lesions. **(S3a-b)** Representative diaphragmatic colonization lesions **(S3a)** and mesenteric colonization lesions **(S3b)** in the NC and MEF2A KD groups. **(S3c-d)** Representative image of diaphragmatic **(S3c)** and mesenteric **(S3d)** colonization lesions and HE staining of these lesions in the control and MEF2A OE groups. Magnification: 100×. Scale bar: 100 µm.

**Figure S4.** **MEF2A expression was positively correlated with that of EMT-related genes. (S4a)** Volcano plot showing the number of differentially expressed genes based on the microarray data (HCT116-MEF2A cells vs HCT116-vec cells). **(S4b)** The correlation between MEF2A expression and ZEB2 or *CTNNB1* expression in CRC samples from the TCGA database.

**Figure S5.** **ZEB2 downregulation inhibited cell migration and invasion but not proliferation. (S5a-b)** qPCR and western blotting showing siRNA-induced ZEB2 knockdown efficiency. **(S5c-d)** The MTS and Transwell assays were used to measure the effect of ZEB2 on cell proliferation, migration and invasion. The experiments were independently repeated three times. **P < 0.01, ***P < 0.001.

**Table S1.** Gene sets for GSEA analysis.
