# Supplementary figures and images for: MEF2A transcriptionally upregulates the expression of ZEB2 and CTNNB1 in colorectal cancer to promote tumor progression
Source: Oncogene. 2021 Apr 16;40(19):3364–77. doi: 10.1038/s41388-021-01774-w (PMC8116210; doi:10.1038/s41388-021-01774-w)

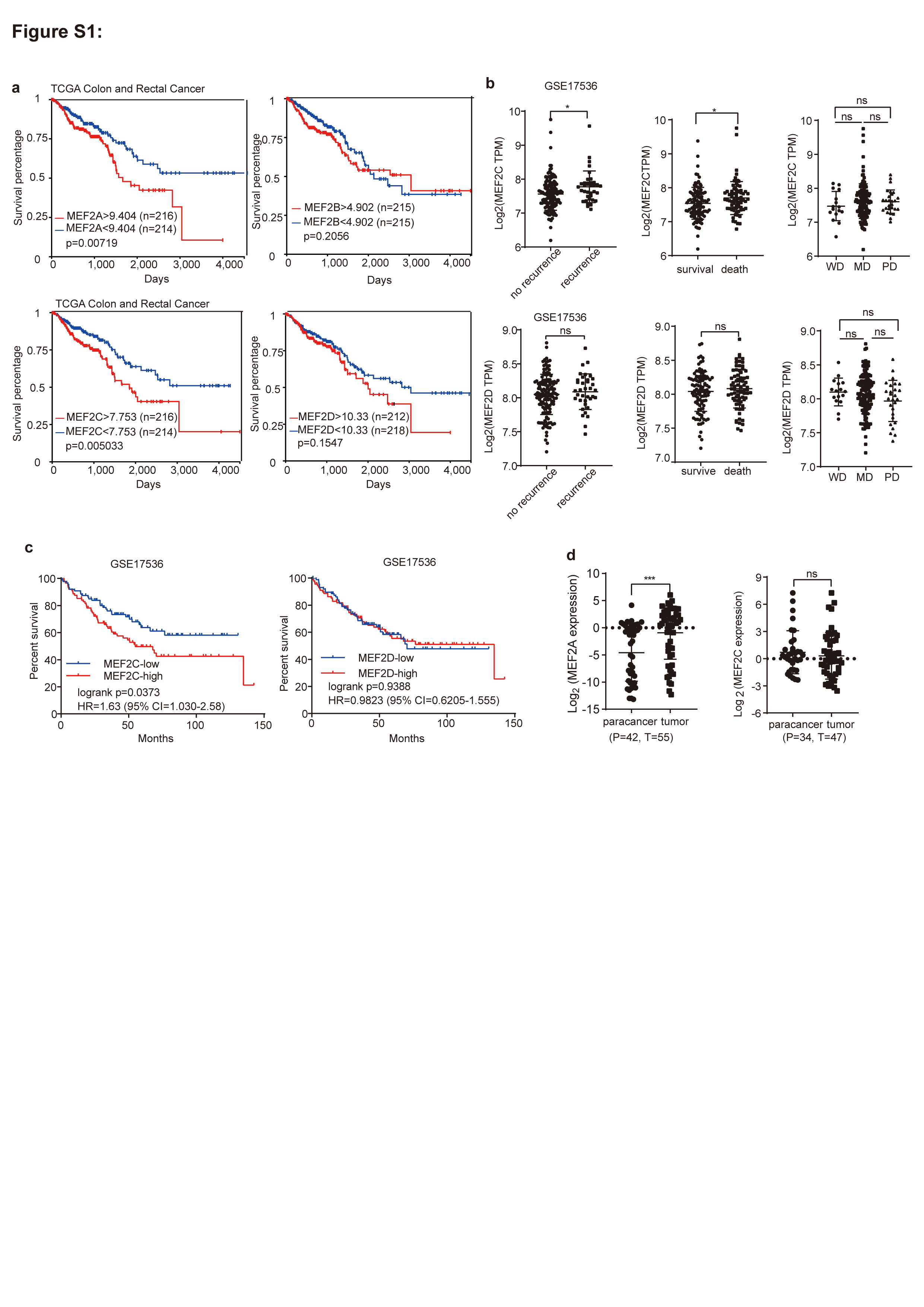

Supplement: Supplementary file 3 — Figure S1 [file 41388_2021_1774_MOESM3_ESM.tif]

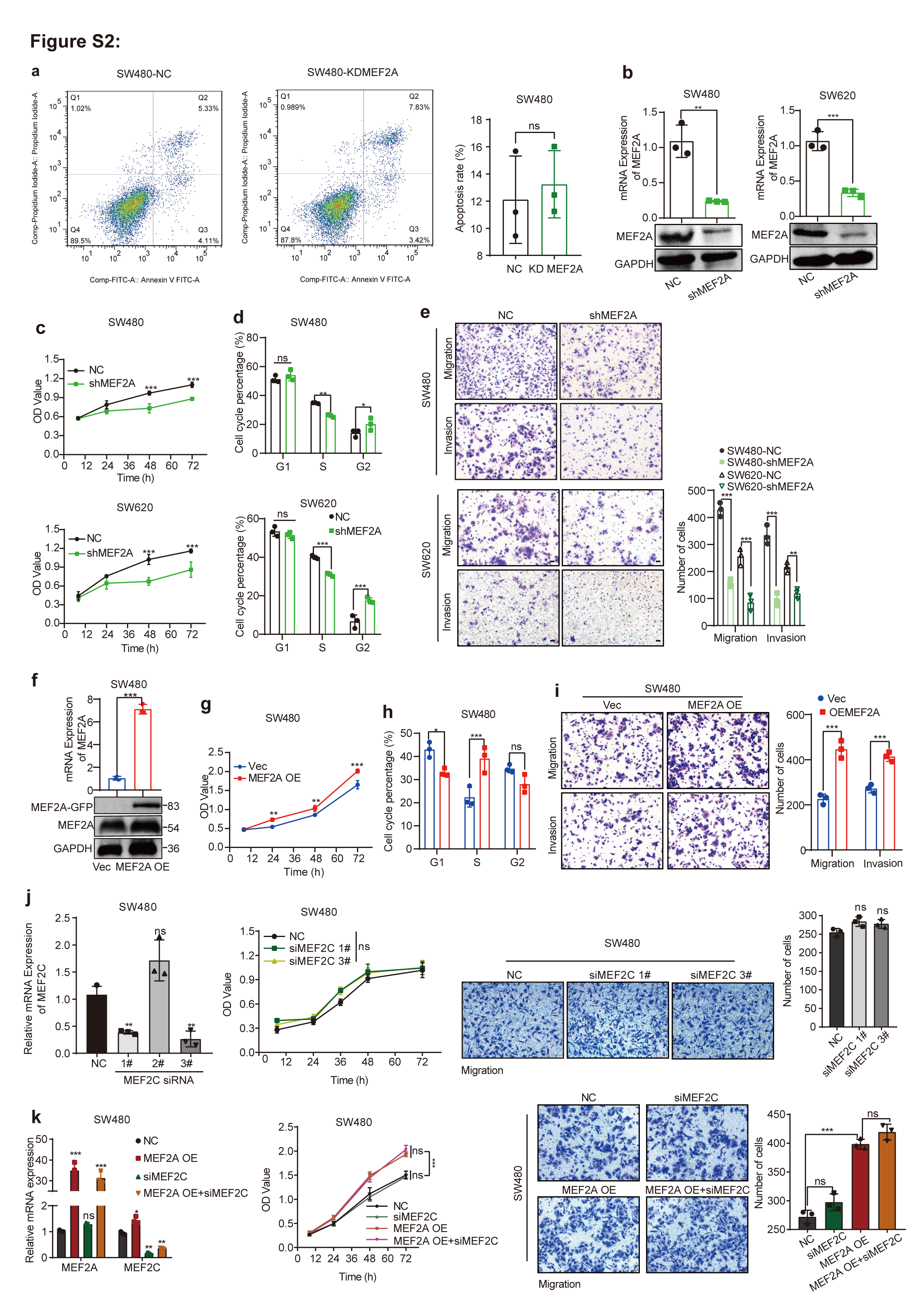

Supplement: Supplementary file 4 — Figure S2 [file 41388_2021_1774_MOESM4_ESM.tif]

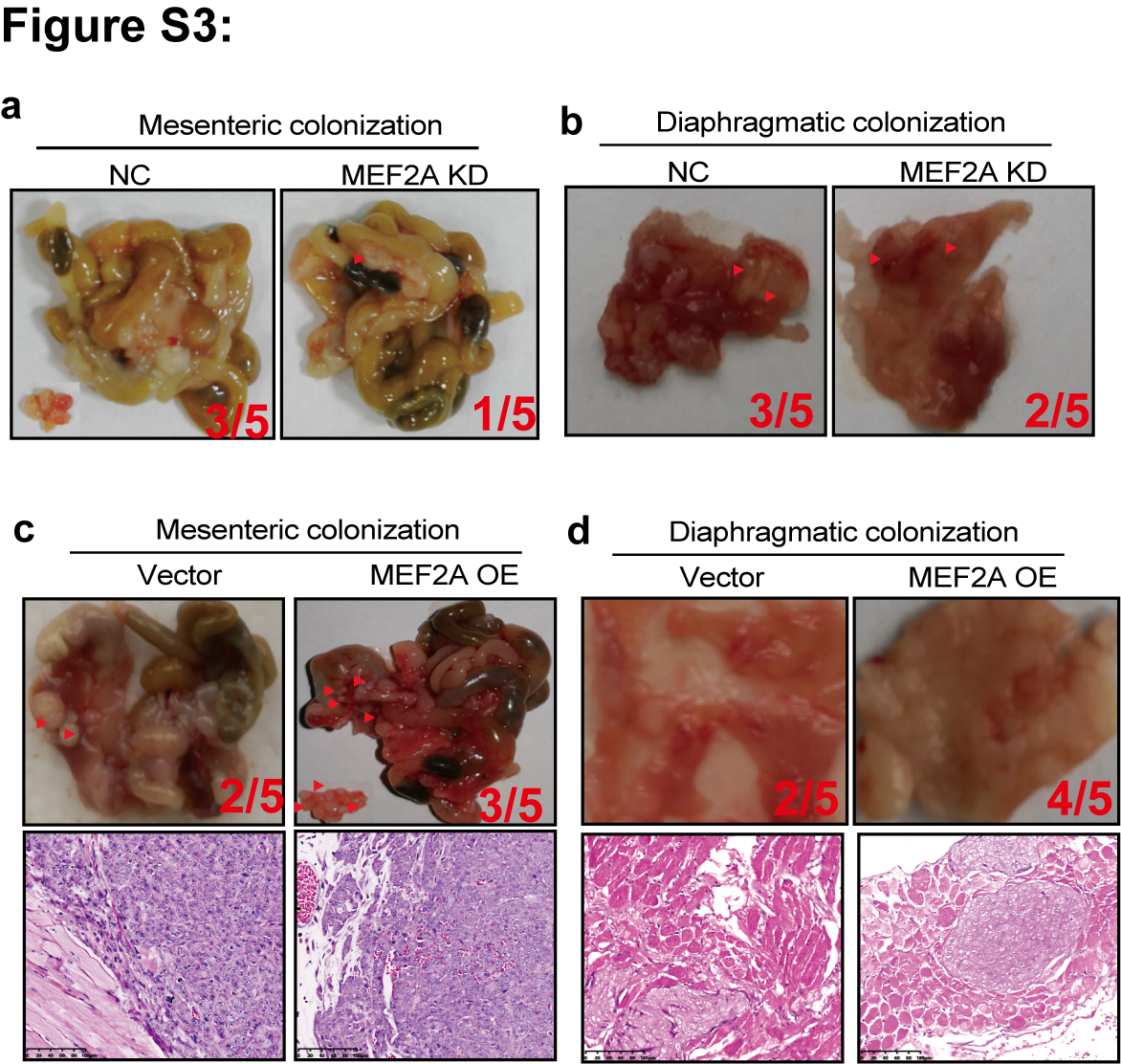

Supplement: Supplementary file 5 — Figure S3 [file 41388_2021_1774_MOESM5_ESM.tif]

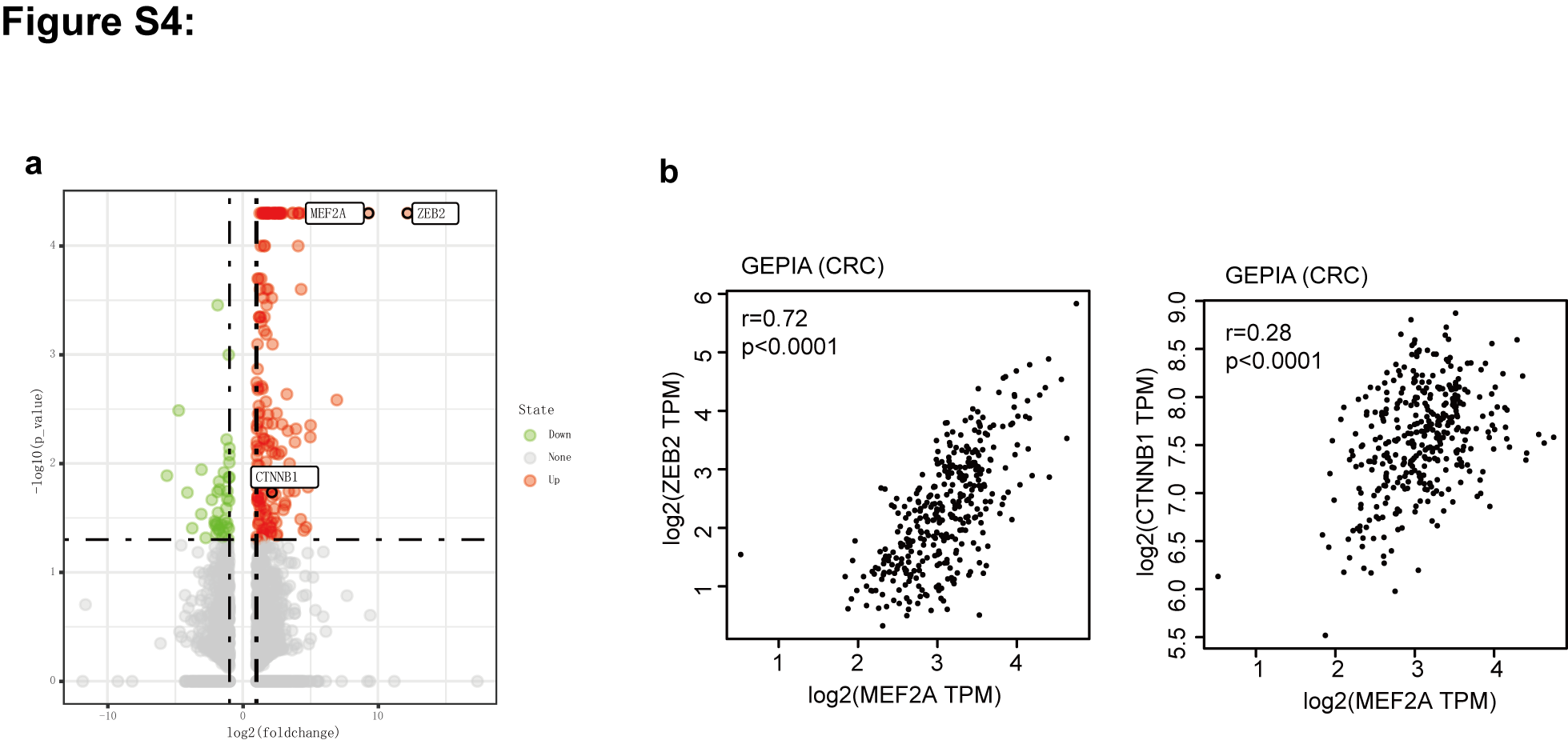

Supplement: Supplementary file 6 — Figure S4 [file 41388_2021_1774_MOESM6_ESM.tif]

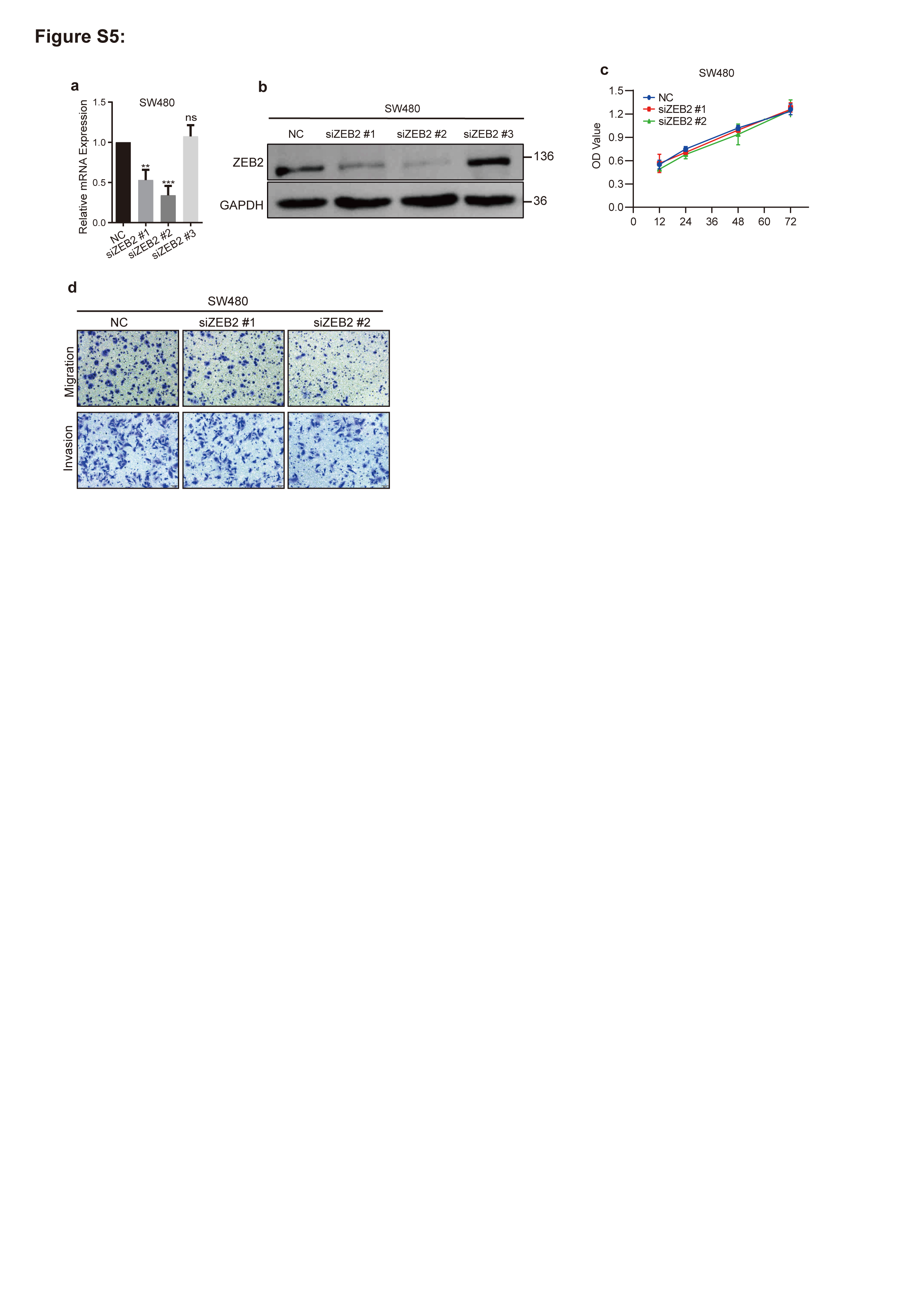

Supplement: Supplementary file 7 — Figure S5 [file 41388_2021_1774_MOESM7_ESM.tif]
